# β-1,3-Glucan recognition by *Acanthamoeba castellanii* as a putative mechanism of amoeba-fungal interactions

**DOI:** 10.1128/aem.01736-23

**Published:** 2024-01-23

**Authors:** Marina da Silva Ferreira, Diego de Souza Gonçalves, Susana Ruiz Mendoza, Gabriel Afonso de Oliveira, Bruno Pontes, Claudia Rodríguez-de la Noval, Leandro Honorato, Luis Felipe Costa Ramos, Fábio C. S. Nogueira, Gilberto B. Domont, Arturo Casadevall, Leonardo Nimrichter, Jose Mauro Peralta, Allan J. Guimaraes

**Affiliations:** 1 Laboratório de Bioquímica e Imunologia das Micoses, Departamento de Microbiologia e Parasitologia, Instituto Biomédico, Universidade Federal Fluminense, Niterói, Rio de Janeiro, Brazil; 2 Programa de Pós-Graduação em Imunologia e Inflamação, Instituto de Microbiologia Paulo de Góes, Universidade Federal do Rio de Janeiro, Niterói, Rio de Janeiro, Brazil; 3 Programa de Pós-Graduação em Doenças Infecciosas e Parasitárias, Faculdade de Medicina, Universidade Federal do Rio de Janeiro, Niterói, Rio de Janeiro, Brazil; 4 Instituto de Ciências Biomédicas e Centro Nacional de Biologia Estrutural e Bioimagem, Universidade Federal do Rio de Janeiro, Niterói, Rio de Janeiro, Brazil; 5 Laboratório de Glicobiologia de Eucariotos, Departamento de Microbiologia Geral, Instituto de Microbiologia Paulo de Góes, Universidade Federal do Rio de Janeiro, Niterói, Rio de Janeiro, Brazil; 6 Departamento de Bioquímica, Instituto de Química, Universidade Federal do Rio de Janeiro, Niterói, Rio de Janeiro, Brazil; 7 Department of Molecular Microbiology and Immunology, Johns Hopkins Bloomberg School of Public Health, Baltimore, Maryland, USA; 8 Rede Micologia RJ - Fundação de Amparo à Pesquisa do Estado do Rio de Janeiro (FAPERJ), Niterói, Rio de Janeiro, Brazil; 9 Pós-Graduação em Microbiologia e Parasitologia Aplicadas, Instituto Biomédico, Universidade Federal Fluminense, Niterói, Rio de Janeiro, Brazil; Centers for Disease Control and Prevention, Atlanta, Georgia, USA

**Keywords:** *Acanthamoeba castellanii*, macrophage, interaction, *Histoplasma capsulatum*, β-glucan

## Abstract

In this study, we conducted an in-depth analysis to characterize potential *Acanthamoeba castellanii* (*Ac*) proteins capable of recognizing fungal β-1,3-glucans. *Ac* specifically anchors curdlan or laminarin, indicating the presence of surface β-1,3-glucan-binding molecules. Using optical tweezers, strong adhesion of laminarin- or curdlan-coated beads to *Ac* was observed, highlighting their adhesive properties compared to controls (characteristic time τ of 46.9 and 43.9 s, respectively). Furthermore, *Histoplasma capsulatum* (*Hc*) G217B, possessing a β-1,3-glucan outer layer, showed significant adhesion to *Ac* compared to a *Hc* G186 strain with an α-1,3-glucan outer layer (τ of 5.3 s vs τ 83.6 s). The addition of soluble β-1,3-glucan substantially inhibited this adhesion, indicating the involvement of β-1,3-glucan recognition. Biotinylated β-1,3-glucan-binding proteins from *Ac* exhibited higher binding to *Hc* G217B, suggesting distinct recognition mechanisms for laminarin and curdlan, akin to macrophages. These observations hinted at the β-1,3-glucan recognition pathway’s role in fungal entrance and survival within phagocytes, supported by decreased fungal viability upon laminarin or curdlan addition in both phagocytes. Proteomic analysis identified several *Ac* proteins capable of binding β-1,3-glucans, including those with lectin/glucanase superfamily domains, carbohydrate-binding domains, and glycosyl transferase and glycosyl hydrolase domains. Notably, some identified proteins were overexpressed upon curdlan/laminarin challenge and also demonstrated high affinity to β-1,3-glucans. These findings underscore the complexity of binding via β-1,3-glucan and suggest the existence of alternative fungal recognition pathways in *Ac*.

**IMPORTANCE:**

*Acanthamoeba castellanii* (*Ac*) and macrophages both exhibit the remarkable ability to phagocytose various extracellular microorganisms in their respective environments. While substantial knowledge exists on this phenomenon for macrophages, the understanding of *Ac*’s phagocytic mechanisms remains elusive. Recently, our group identified mannose-binding receptors on the surface of *Ac* that exhibit the capacity to bind/recognize fungi. However, the process was not entirely inhibited by soluble mannose, suggesting the possibility of other interactions. Herein, we describe the mechanism of β-1,3-glucan binding by *A. castellanii* and its role in fungal phagocytosis and survival within trophozoites, also using macrophages as a model for comparison, as they possess a well-established mechanism involving the Dectin-1 receptor for β-1,3-glucan recognition. These shed light on a potential parallel evolution of pathways involved in the recognition of fungal surface polysaccharides.

## INTRODUCTION

*Acanthamoeba* spp. has been detected in various environments, including soil, air, lakes, contact lens solutions, and even in water intended for human consumption (1–5). The rise in reported cases of *Acanthamoeba* infections in the human population has been directly linked to the more frequent isolation/detection of this species in the environment (6, 7). An additional emerging concern is the consistent observation of endosymbiont pathogens when *Acanthamoeba* spp. are isolated from environmental sources (4, 5). Nevertheless, these amoebae not only pose an increasing public health risk but also serve as potential reservoirs for significant human pathogens, including viruses, bacteria, and endemic fungi (4, 8–12). Hence, further investigations are warranted to comprehend the intricate web among different organisms by studying the association of free-living amoebae (FLA) with their environment, including interactions with classes of pathogens that inhabit the same niches (4, 11–15).

In soil, amoebae encounter various adverse conditions such as fluctuations in pH, oxygen levels, extreme temperatures, UV radiation, nutrient competition, and predation by other competing organisms (5, 16). Predation is also a mechanism used by amoebae for nutrient acquisition and survival, and they presumably possess a repertoire of receptors or molecules capable of recognizing a myriad of microorganisms in the environment (4, 5, 17, 18). In the literature, receptors of *Acanthamoeba castellanii* (*Ac*) (16) for bacteria (19) and viruses (20) have been described as lectin-like transmembrane proteins capable of recognizing polysaccharides on the surface of these microorganisms (4, 5, 21, 22).

Analysis of the ribosomal RNA sequences reveals that protists like *Acanthamoeba* exhibit a substantial genetic similarity with fungi and animals, indicating their potential for phagocytosis, movement, and adaption in ways similar to human phagocytic cells (23, 24). Moreover, amoebae demonstrate the ability to distinguish between self and non-self-molecules through a mechanism analogous to that employed by cells from the immune system of various organisms (25). Specifically, *Acanthamoeba* spp. shares several characteristics with human macrophages, including morphological, biochemical, physiological, and behavioral particularly in their interaction with other microorganisms. Some authors hypothesize that amoebae and macrophages share similar mechanisms of interaction, potentially involving cellular receptors on their surfaces that play a critical role in the phagocytic activity of amoebae (24, 26, 27).

Recently, our research team has successfully identified two mannose-binding proteins (MBPs) from *Ac* that possess the remarkable capacity to bind and recognize mannose residues on the fungal cell walls (21, 22). These MPBs could potentially work as “universal” receptors, mediating the phagocytosis of several pathogenic and non-pathogenic fungi. Nonetheless, it was observed that soluble mannose only resulted in approximately 70% reduction of phagocytosis without complete inhibition, implying the plausible involvement of additional receptors in mediating the intricate fungi-amoeba interactions (4, 21, 22). Furthermore, mannose recognition seems to be a conserved mechanism for fungal recognition, adaptation, and survival among various phagocytes, including amoeba and macrophages (28).

Macrophages can recognize β-1,3-glucans present on the fungal cell walls, through the phagocytic Dectin-1 receptor, a C-type lectin that triggers internalization pathways in response to the pathogen (29, 30). Our results have unequivocally demonstrated the ability of *A. castellanii* to bind β-1,3-glucan on the surface of pathogenic fungi, as an alternative mechanism of recognition or possibly as a synergistic action with other receptors involved in amoeba-fungal attachment. These findings provide robust support for the hypothesis of additional conserved mechanisms for fungal recognition, thereby implying a potential common evolutionary origin between the two natural phagocytes, *A. castellanii* and macrophages.

## RESULTS

### *A. castellanii* express surface ligands able to recognize β-1,3-glucan

**Fig 1:**
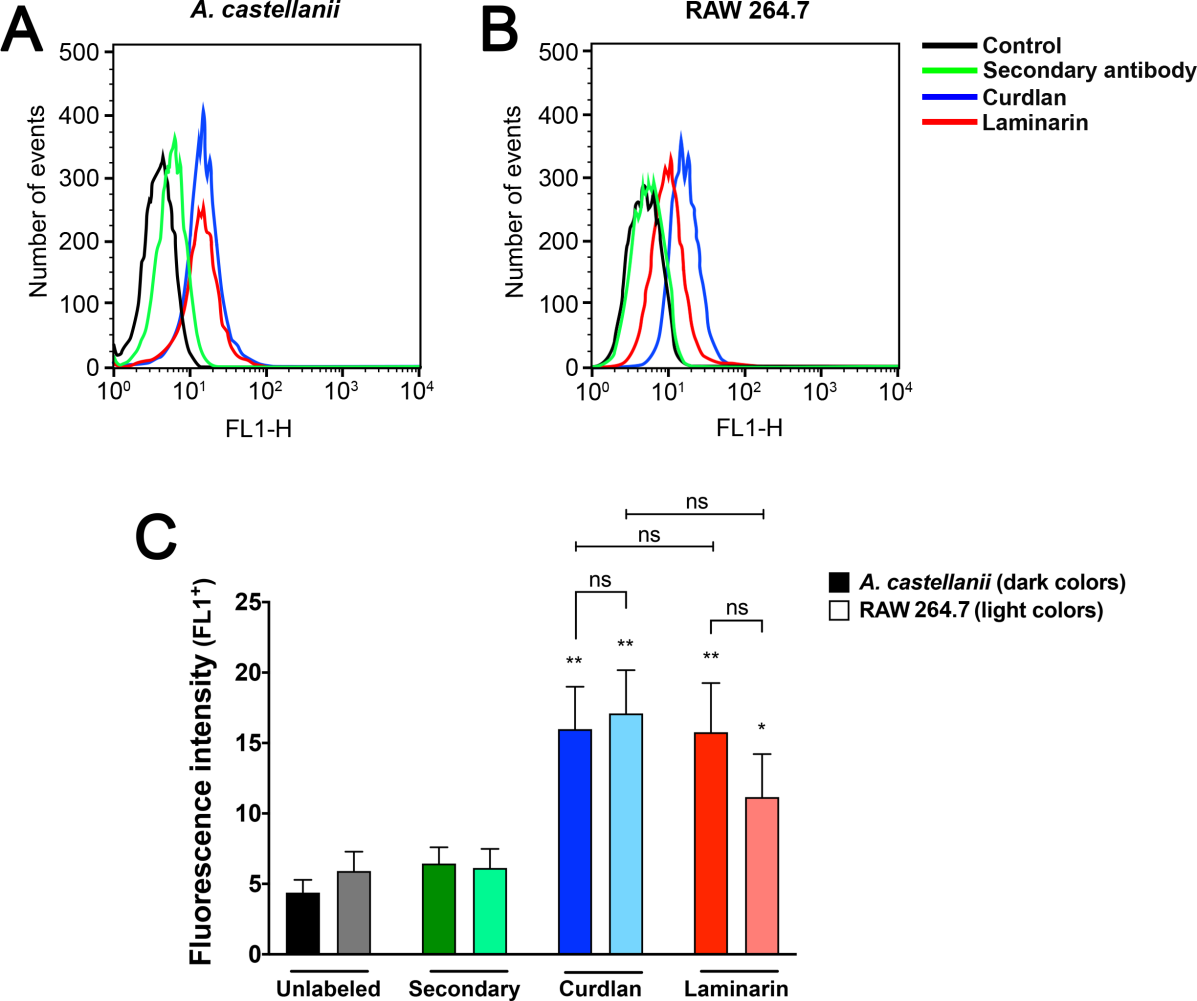
Detection of β-1,3-glucan binding on the surface of *Acanthamoeba castellanii* and RAW macrophages: trophozoites of *A. castellanii* or RAW macrophages were pre-incubated with curdlan and laminarin for 45 min. After which, phagocytes were fixed and labeled with Dectin-1-Fc/anti-mouse IgG-Alexa 488, and the fluorescence intensity of the FL1+ channel was evaluated by flow cytometry. Fluorescence intensity histograms for both (**A**) *A. castellanii* and (**B**) RAW cells were plotted. (**C**) The fluorescence means of laminarin and curdlan of both phagocyte’s models were compared to controls (*P < 0.05), internally within each phagocyte (*P* < 0.05) and between *A. castellanii* (dark colors) and RAW macrophages (light colors). Graphs represent the average of three independent experiments (*P < 0.05, **P < 0.01, and ns, not significant).

Recognition of β-1,3-glucan by *Ac* and RAW macrophages was initially assessed by flow cytometry (Fig. 1A and B, respectively). Amoebae were pre-incubated with curdlan and laminarin, followed by their binding detection with Dectin-1-Fc/anti-mouse-Alexa 488. Both curdlan and laminarin bound to the surface of *Ac* trophozoites (Fig. 1A). Specifically, when comparing both polysaccharides, the mean FL1+ intensity values were 16.0 and 15.8 respectively, which were higher than unlabeled controls (4.4) and secondary antibody controls in the absence of primary antibody (6.4; Fig. 1C, *P < 0.05). For the RAW macrophages, the incorporation of curdlan (17.1) displayed higher fluorescence values compared to laminarin (11.2; Fig. 1B and C), and both polysaccharides displayed higher labeling values than the unlabeled (5.9) and secondary (6.1) controls. Relative comparison between the phagocytes demonstrated similar curdlan and laminarin binding in both models (ns, not significant; Fig. 1C).

### β-1,3-Glucan affinity determination of *A. castellanii* biotinylated surface proteins in comparison to macrophages

**Fig 2:**
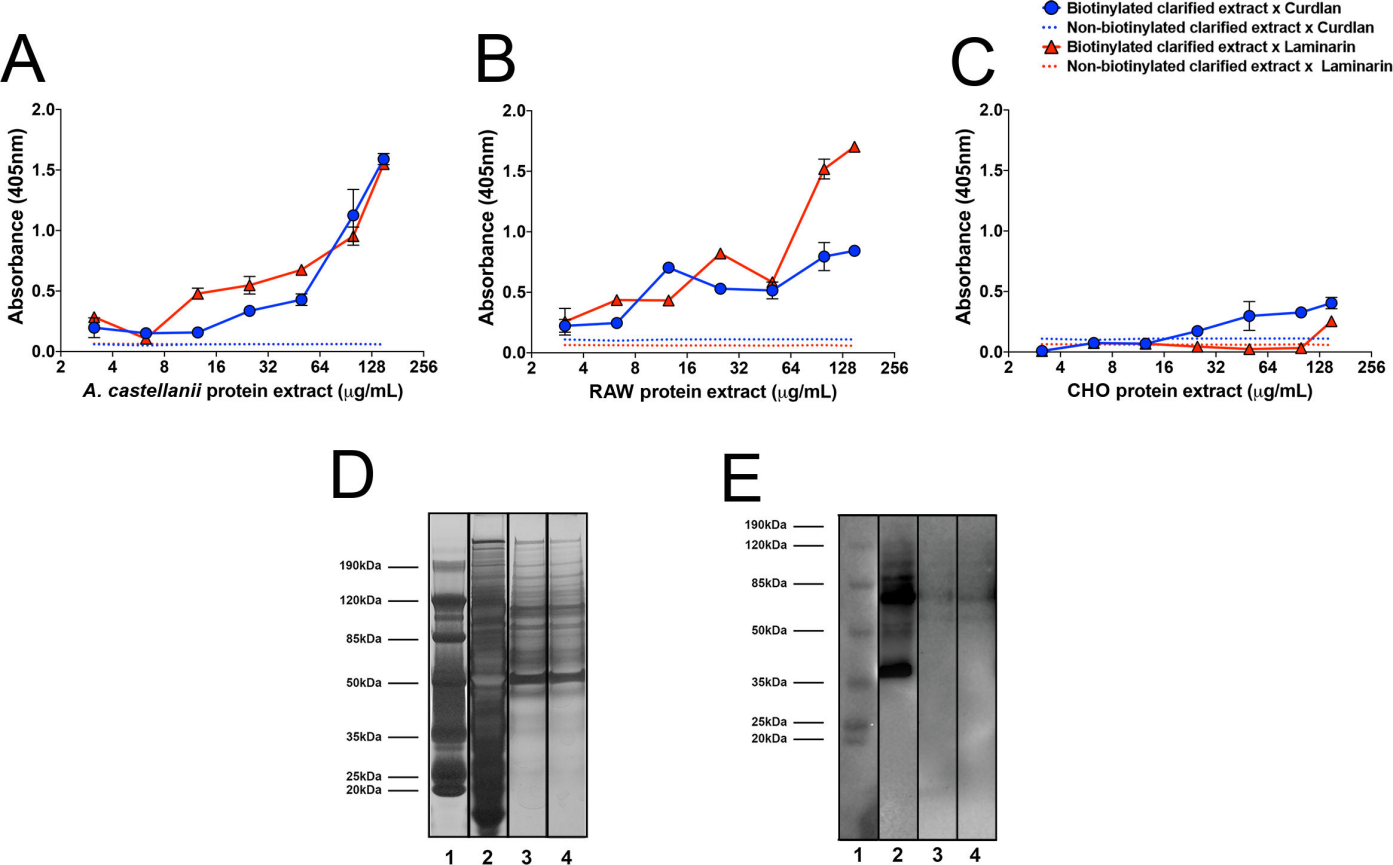
Binding evaluation of distinct cell line’s biotinylated surface extracts to β-1,3-glucans. Curdlan (blue curves) and laminarin (red curves) polysaccharides were immobilized at the concentration of 10 µg/mL in 96-well plates. The biotinylated membrane extracts from (**A**) *Acanthamoeba castellanii,* (**B**) RAW macrophages, and (**C**) CHO were incubated for 1 h at 37 °C at protein concentrations ranging from 150 µg/mL to 3.125 µg/mL. A streptavidin phosphatase alkaline conjugate (1:2,000) was used for the detection of biotinylated proteins, for 1 h at 37°C, and plated read at 450 nm. (**D**) SDS-PAGE for the detection of surface biotinylated protein extract of *A. castellanii* with affinity to either curdlan or laminarin. Lane 1, protein weight mass standard (BenchMark Pre-stained protein Ladder; Thermo Fisher); lane 2, total surface biotinylated proteins extract of *A. castellanii* (total); lane 3, proteins stripped after binding to curdlan; lane 4, proteins stripped after binding to laminarin. (**E**) Western blot (WB) for the detection of biotinylated surface proteins of *A. castellanii* with affinity to β-1,3-glucans. Lane 1, BenchMark Pre-stained Protein Ladder; Thermo Fisher); lane 2, total surface biotinylated proteins extract of *A. castellanii* (total); lane 3, biotinylated proteins of *A. castellanii* with affinity to curdlan; lane 4, biotinylated proteins of *A. castellanii* with affinity to laminarin.

To evaluate the presence of β-1,3-glucan-binding proteins in the biotinylated surface extracts of *Ac*, we conducted an enzyme-linked immunosorbent assay (ELISA) using microplates coated with curdlan or laminarin. The *Ac* biotinylated surface extracts showed a dose-dependent binding to laminarin and curdlan, with no significant differences comparing their reactivity (Fig. 2A, *P* > 0.05). As control systems, we compared the binding of the extracts to the polysaccharides using Dectin-1 expressing RAW 264.7 macrophages (Fig. 2B) and Dectin-1 absent Chinese hamster ovary (CHO) epithelial cells (Fig. 2C). Biotinylated extract from RAW 264.7 exhibited higher reactivity to laminarin than curdlan, but only at concentrations >50 µg/mL (Fig. 2B, *P* = 0.0009). In contrast, biotinylated CHO extracts showed insignificant or no binding to any of the polysaccharides used in our analysis. As expected, non-biotinylated mock extracts in all the systems displayed no reactivity.

Additionally, we analyzed the biotinylated extract of *Ac* by SDS-PAGE and Western blot (WB) to evaluate the profile of proteins with binding capacity to β-1,3-glucan-coated microplates. The total extract prior to incubation with either curdlan or laminarin revealed a pool of proteins ranging from ~250 kDa to smaller molecular weight proteins (<20 kDa; Fig. 2D, lane 2). A significant number of proteins with affinity to curdlan (Fig. 2D, lane 3) or laminarin (Fig. 2D, lane 4) were observed, displaying similar profiles upon detachment with stripping buffer after binding to the polysaccharides. However, WB analysis using streptavidin-alkaline phosphatase specifically detected seven visible bands, representing biotinylated surface proteins with affinity to β-1,3-glucans, with molecular weights ranging from 50 to 125 kDa (~125, 112, 100, 90, 75, 60, and 52.5 kDa) for both curdlan (Fig. 2E, lane 3) and laminarin (Fig. 2E, lane 4).

### β-1,3-Glucan-coated polystyrene beads quickly interact with the surface of *A. castellanii*

**Fig 3:**
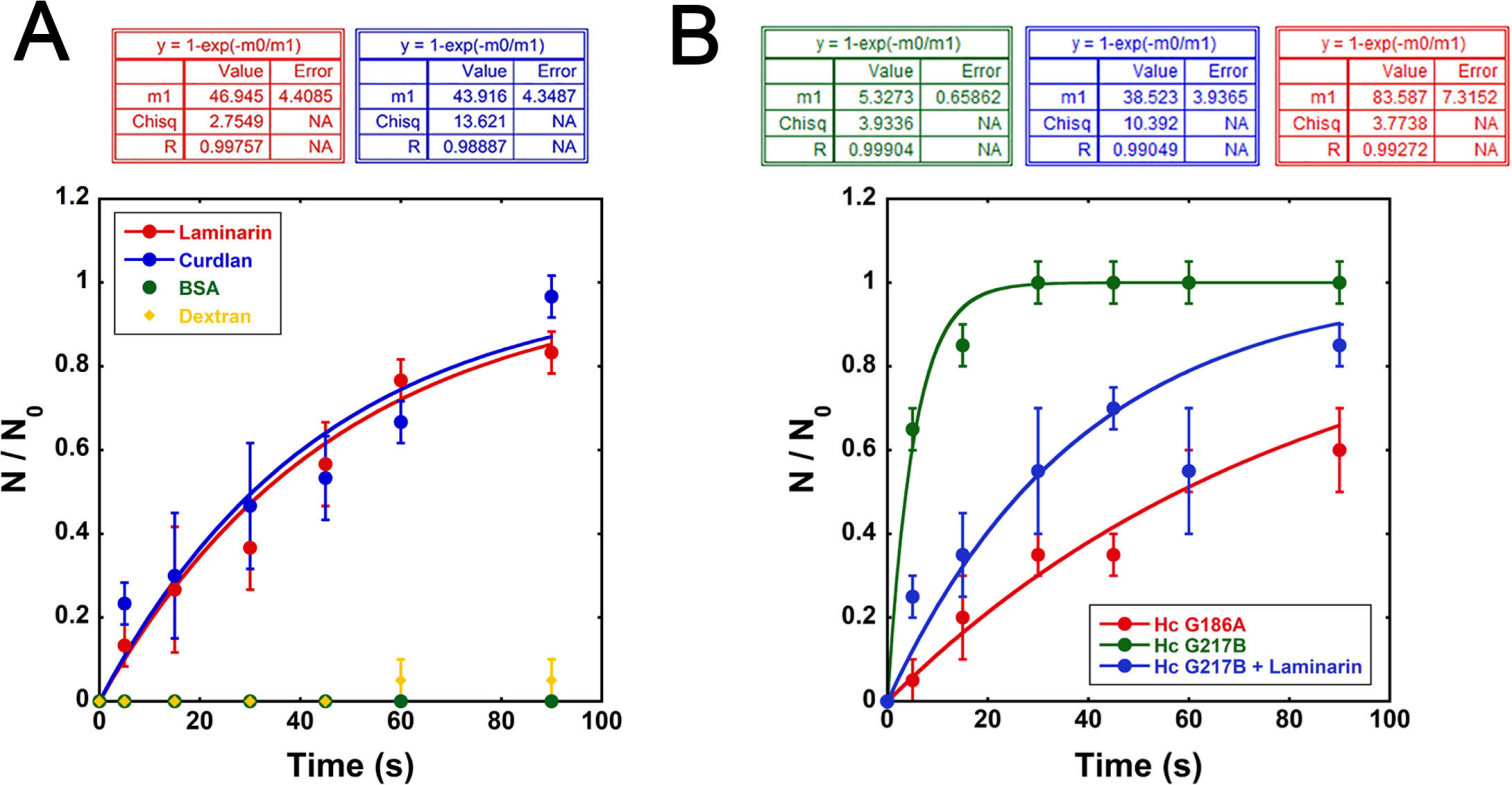
Optical tweezers-based assay to evaluate the adhesion of glucans to *Acanthamoeba castellanii*. (**A**) β-1,3-Glucan-coated polystyrene beads, curdlan (blue curves), or laminarin (red curves) were captured by a laser and allowed to interact with the surface of *A. castellanii*. BSA (green circles) or dextran-coated polystyrene beads (yellow diamonds) were used, with insignificant interactions occurring. (**B**) *Histoplasma capsulatum* (*Hc*) yeasts were also captured by optical tweezers and allowed to interact with *Ac. Hc* G217B, which expresses a β-1,3 glucan as the most external layer, interacted strongly with *Ac* (green curve). The presence of an α-1,3-glucan external layer on the *Hc* G186A strain offered a steric hindrance of β-1,3-glucan recognition by *Ac* and strongly impaired the adhesion capacity of *H. capsulatum* (red circle). Interaction specificity through β-1,3 glucan in our experiments was confirmed by adding soluble laminarin during *Hc* G217B-*Ac* interactions, which inhibited the *Hc-Ac* adhesion (blue circle).

Optical tweezers are excellent tools for assessing real-time interactions between microscopic size structures. Polystyrene beads, previously coated with curdlan or laminarin, manifested notable adhesion to the surface of *Ac* (Movie S1). The best curve fits were plotted, and the calculated characteristic times [τ] *f*or both β-1,3-glucans were remarkably similar, measuring 43.9 ± 4.3 s and 46.9 ± 4.4 s, respectively (*P* > 0.05; Fig. 3A, blue and red curves). As negative controls, bovine serum albumin (BSA) or dextran-coated beads displayed negligible or insignificant adhesion (Fig. 3A, green circles and yellow diamonds).

To confirm the recognition of fungal β-1,3-glucan by *Ac*, we employed a *Histoplasma capsulatum* (*Hc*) G217B strain expressing exposed β-1,3-glucan on the cell wall and compared it to an *Hc* G186A strain, which has an external α-1,3-glucan layer over the β-1,3-glucan (31). Strikingly, the *Hc* G217B yeast rapidly attached to the *Ac* surface, with a characteristic time τ of 5.3 ± 0.6 s (Fig. 3B, green curve). In contrast, the association of the *Hc* G186A strain with *Ac* was significantly impaired, displaying a characteristic time τ of 83.6 ± 7.3 s (*P* < 0.05, Fig. 3B, red curve). Moreover, to validate the specificity of interactions with β-1,3-glucan within the proposed system, pre-incubation of *Ac* with laminarin increased the characteristic time τ from 5.3 ± 0.6 s to 38.5 ± 3.9 s (*P* < 0.05, Fig. 3B, green compared to blue curve).

### *A. castellanii* biotinylated surface β-1,3-glucan-binding proteins accessibility correlated to higher *A. castellanii-fungus* interaction rates

**Fig 4:**
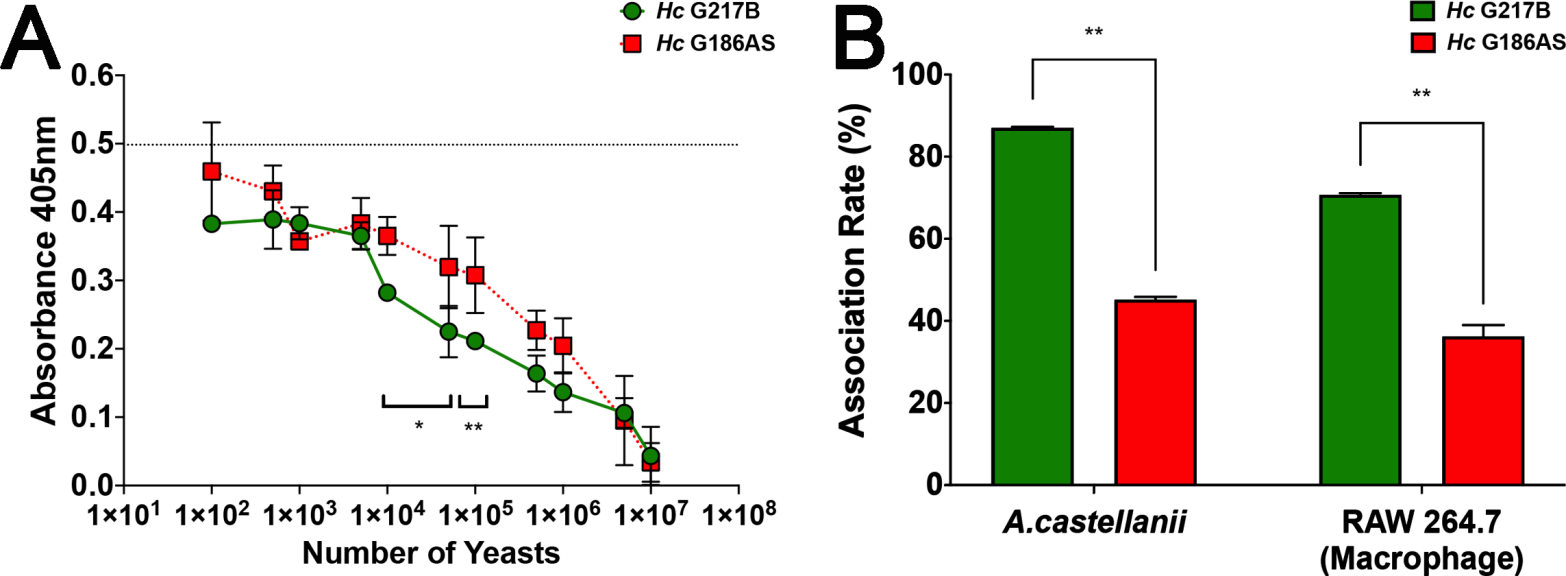
*A. castellanii* biotinylated surface β-1,3-glucan-binding proteins affinity to fungi and the association rates of two different strains of *H. capsulatum*. (**A**) Biotinylated surface β-1,3-glucan-binding proteins of *A. castellanii* bound to *Hc* G217B (green curve) and *Hc* G186A (red curve) yeasts evaluated in this study in a dose-dependent fashion; overall higher inhibition was detected by the *Hc* G217B strain, with lower levels of remaining β-1,3-glucan-binding proteins and ligation to the immobilized laminarin on the reaction plate (*P < 0.05, **P < 0.01). The dashed line indicates the maximum binding of the *A. castellanii* biotinylated surface β-1,3-glucan-binding proteins in the absence of yeast inhibition. (**B**) Interaction between *A. castellanii* and RAW with *Hc* G217B (green bars) and *Hc* G186A (red bars). Yeasts were labeled with NHS-Rhodamine for 1 h, washed, and submitted to interact with *Ac* (28°C) or RAW (37°C/ 5% CO_2_) for 2 h at a multiplicity of infection of 2:1. Systems were read by flow cytometry and association rates were calculated.

An inhibition ELISA using the β-1,3-glucan-binding biotinylated surface membrane extract of *Ac* revealed a dose-dependent inhibition upon incubations with the different *Hc* strains. The *Hc* G217B strain exhibited the most pronounced inhibition, indicating greater ligation of β-1,3-glucan affinity protein to these yeasts and subsequently reduced detection of free-β-1,3-glucan affinity protein binding to laminarin on the reaction plates (Fig. 4A). Similarly, the *Hc* G186A strain showed a dose-dependent inhibition, albeit less intense due to its α-1,3-glucan composition (*P* < 0.05).

Building on the confirmatory nature of the above analyses, interaction assays were performed with both *Hc* G217B and *Hc* G186A strains against *Ac* trophozoites, with RAW macrophages serving as controls (Fig. 4B). *Hc* G217B displayed a higher association rate to both *Ac* and RAW macrophages (87% and 71%, respectively), about twofold greater than *Hc* G186A association to either phagocyte (45% and 36%, respectively, *P* < 0.01).

### *A. castellanii* biotinylated surface β-1,3-glucan-binding proteins bound to the surface of *H. capsulatum* and co-localized with Dectin-1-Fc

**Fig 5:**
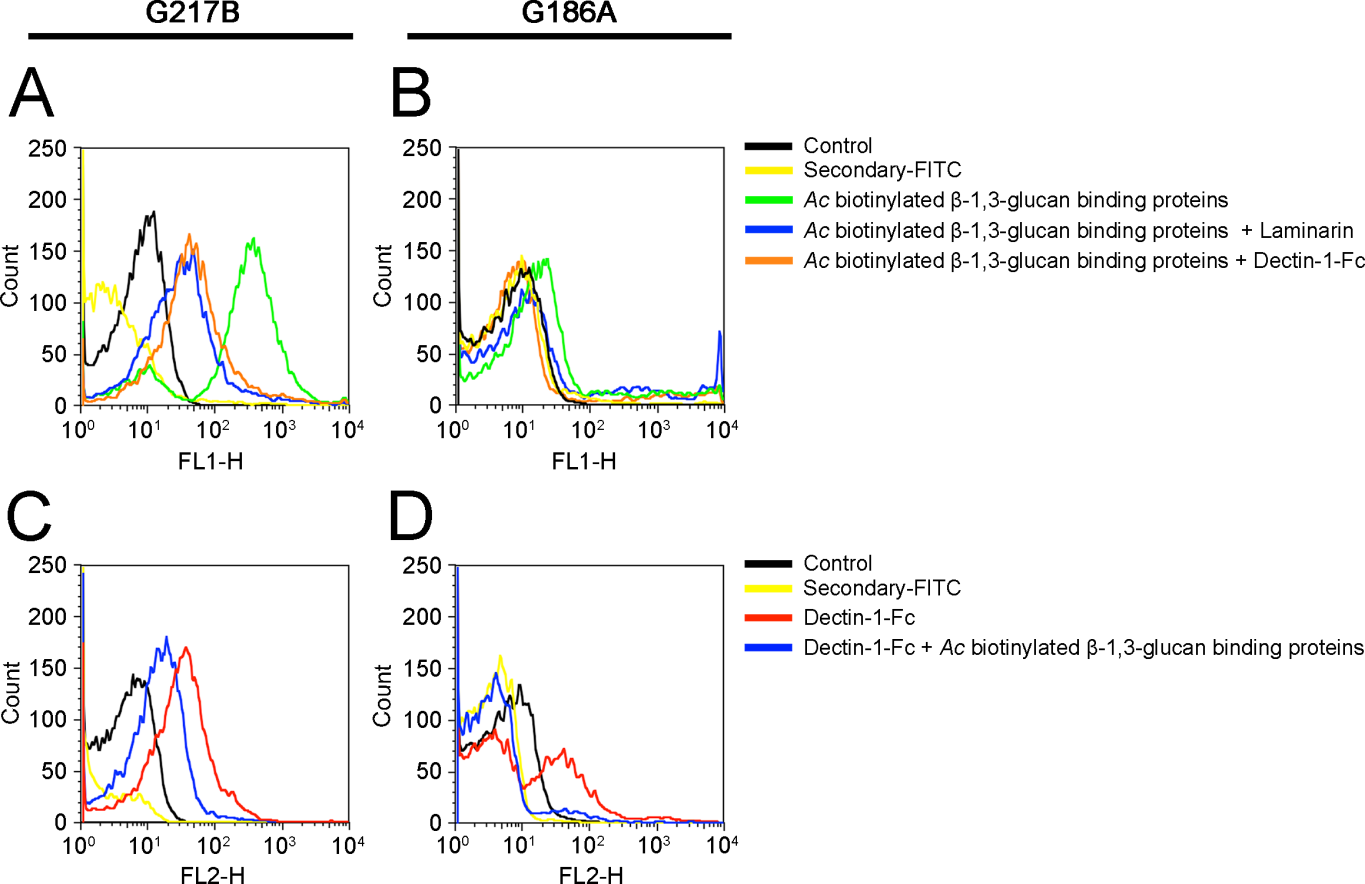
Characterization of the surface β-1,3-glucan-binding proteins of *A. castellanii* binding to the surface of *Histoplasma capsulatum*. (**A–D**) Flow cytometry displaying the binding of biotinylated surface β-1,3-glucan-binding proteins of *Ac* and a streptavidin-Fluorescein (FITC) conjugate (FL1-H) to (**A**) *Hc* G217B and (**B**) *Hc* G186A and (**C and D**) controls of Dectin-1-Fc/anti-mouse IgG-Alexa 546 labeling (FL2-H) to (**C**) *Hc* G217B and (**D**)*Hc* G186A. *Hc* G217B yeasts were strongly labeled with the biotinylated surface β-1,3-glucan-binding proteins of *Ac*, which was inhibited by co-incubations with laminarin or Dectin-1-Fc. In turn, Dectin-1-Fc labeling was inhibited with laminarin or with the biotinylated surface β-1,3-glucan-binding proteins of *Ac*.

Fluorescence-labeling experiments were conducted using the two *Hc* strains, *Hc* G217B and *Hc* G186A, to validate the ligation and specificity of the β-1,3-glucan-binding proteins present in the clarified biotinylated surface extracts of *Ac* (FL1+ fluorescence). Both yeasts were also incubated with Dectin-1-Fc as a labeling control to assess the presence and accessibility of β-1,3-glucan on their surfaces (FL2+). Additionally, the specificity was evaluated through binding inhibition of *Ac* biotinylated surface β-1,3-glucan-binding proteins ligation upon co-incubation with laminarin. The *Hc* G217B yeasts exhibited the highest fluorescence intensity upon binding by the β-1,3-glucan-binding proteins of *Ac* biotinylated surface extracts (Fig. 5A). Conversely, *Hc* G186A demonstrated lower fluorescence, indicative of reduced surface accessibility to β-1,3-glucan (Fig. 5B). In both strains, co-incubations with either laminarin or Dectin-1-Fc hindered the binding of *Ac* biotinylated surface proteins to β-1,3-glucan. Moreover, when *Hc* G217B (Fig. 5C) and *Hc* G186A (Fig. 5D) were incubated with Dectin-1-Fc/anti-mouse-Alexa 546, as a control for β-1,3-glucan accessibility, similar results were observed as with the extracts described above; notably, the binding of Dectin-1-Fc was more prominent to *Hc* G217B than *Hc* G186A. Consequently, co-incubations of Dectin-1-Fc and *Ac* biotinylated surface proteins competed for the binding to β-1,3-glucan in both strains, reducing the overall fluorescence intensity of the FL2+ yeasts.

**Fig 6:**
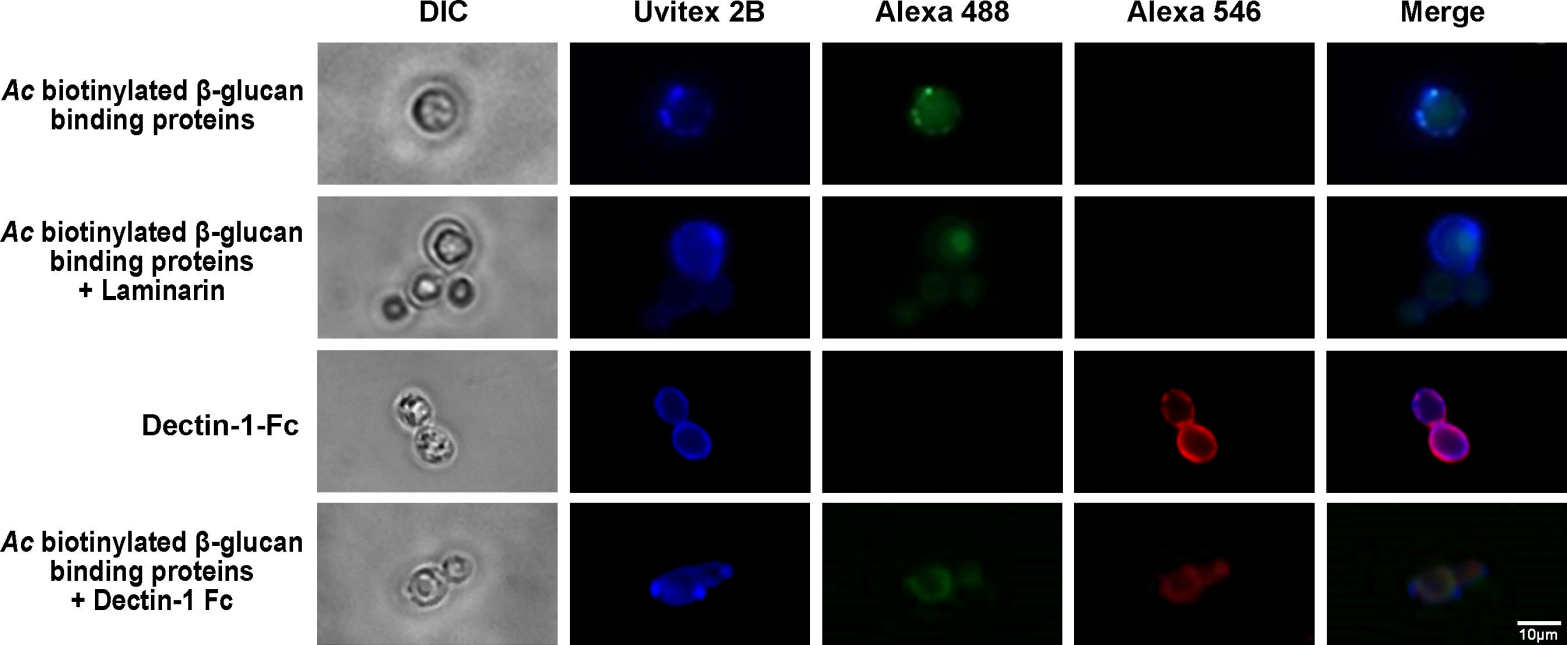
Co-localization of the *A. castellanii* biotinylated surface β-1,3-glucan-binding proteins with Dectin-1-Fc on *H. capsulatum* surface by fluorescence microscopy. Fluorescence microscopy displaying a gear-like labeling by *Ac* biotinylated surface β-1,3-glucan-binding proteins (green fluorescence), which co-localized with the Dectin-1-Fc labeling (red fluorescence). As previously reported, binding was inhibited by laminarin or Dectin-1-Fc; in turn, controls of Dectin-1-Fc labeling were inhibited by laminarin itself or biotinylated surface β-1,3-glucan-binding proteins of *Ac*.

Consistent with the aforementioned results, fluorescence microscopy with the β-1,3-glucan-binding proteins of *Ac* biotinylated surface displayed a gear-like binding pattern on the surface of *Hc* G217B yeasts (Fig. 6, first row), and their binding was inhibited by the addition of soluble laminarin (Fig. 6, second row). As described previously by our group (32), Dectin-1-Fc/IgG-anti-mouse-Alexa 546 labeling of β-1,3-glucans displays a ring-like pattern with randomly located clusters (Fig. 6, third row). As observed in flow cytometry, co-incubations of the β,1,3-glucan-binding proteins of *Ac* biotinylated surface and Dectin-1-Fc led to competition, resulting in reduced binding of either to *H. capsulatum*, as indicated by the lower fluorescence intensity on the yeast surface (Fig. 6, last row). Despite the lower fluorescence, we were able to observe colocalization of the Dectin-1-Fc and β-1,3-glucan-binding proteins labeling on the *Hc* G217B cell wall, indicating the same specificity of both.

### Soluble β-1,3-glucan inhibited the interaction between *A. castellanii* and *H. capsulatum*

**Fig 7:**
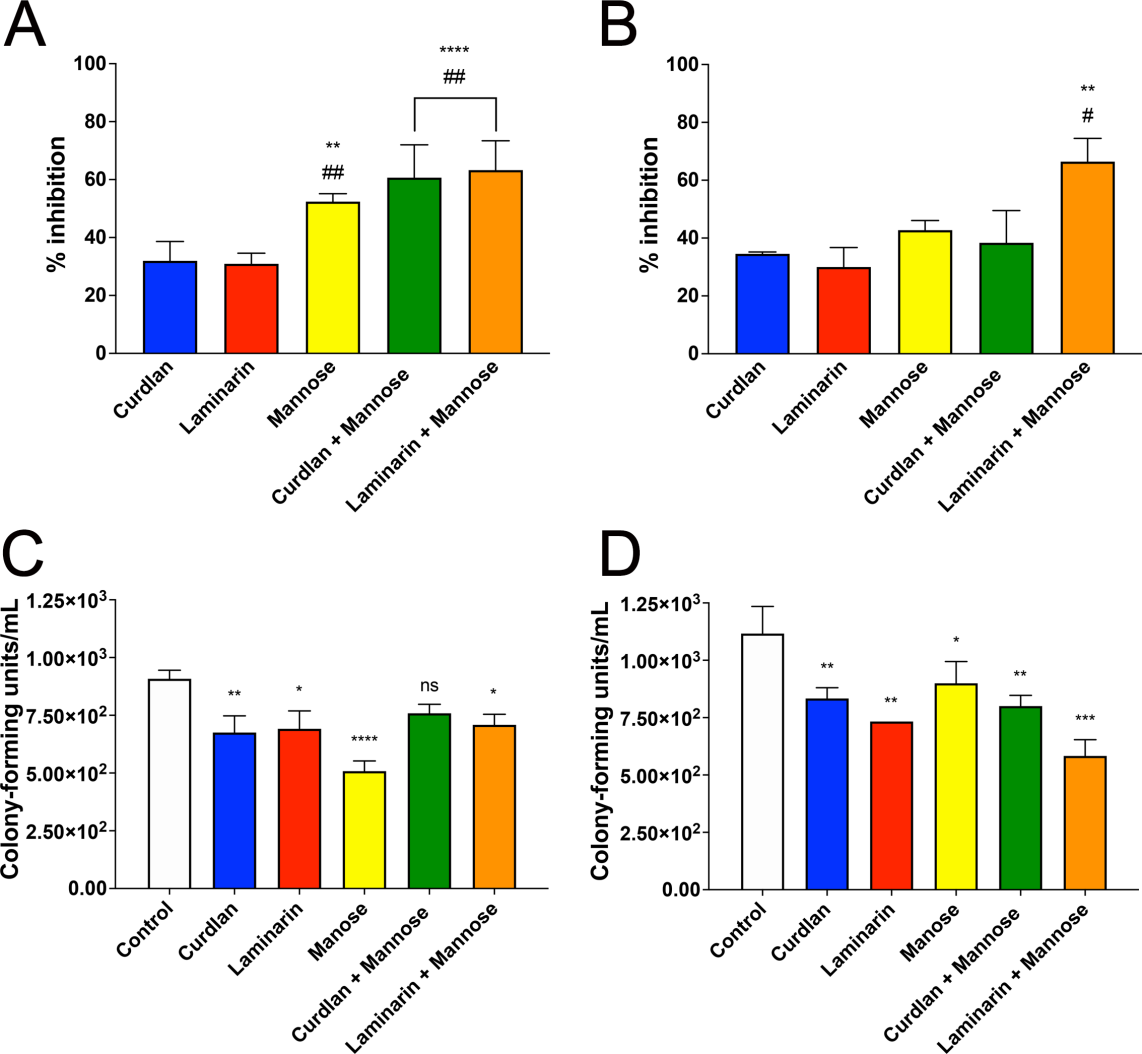
Interaction and survival assay of *H. capsulatum* with *A. castellanii* and macrophages in the presence of different sugars. Relative participation of the β-1,3-glucan-binding protein of *Ac* and RAW 264.7 macrophages on the interaction with *H. capsulatum* G217B and fungal survival. (**A**) *Ac* and (**B**) RAW macrophages were incubated with *Hc* G217B stained with NHS-Rhodamine in the presence of curdlan, laminarin, and mannose (or their combinations), and interactions evaluated by flow cytometry in an FL2-H channel. Fungal killing assay upon overnight incubations of the systems and conditions was also performed and evaluated for the *Hc* G217B interactions with (**C**) *Ac* and (**D**) RAW macrophages (ns, non-significant, *P < 0.05, **P < 0.01, and ***P < 0.001).

In line with previous findings, *Hc* G217B demonstrated the highest interaction rates with both *Ac* and RAW macrophages. To elucidate the requirements of β-1,3-glucan recognition during the interactions, co-incubations with curdlan and laminarin were performed, and combined with mannose, to assess independent mechanisms of recognition for these two polysaccharides. Co-incubations of yeasts with curdlan and laminarin led to inhibitions of *Ac-Hc* G217B interactions by 32.0% and 30.9%, respectively, while the addition of mannose resulted in a 52.4% inhibition (Fig. 7A, *P* < 0.01). Combinations such as mannose plus curdlan or laminarin enhanced the inhibition to 60.7% and 63.8%, respectively (Fig. 7A, *P* < 0.0001). These results suggest that possible receptors capable of recognizing both β-1,3-glucan and mannose on the fungal surface actively contribute to the *Ac-Hc* G217B interaction and may include distinct molecules with independent or overlapping recognition capacity.

A similar assay was performed with *Hc* G217B and RAW macrophages. Employing the same experimental design, co-incubations with curdlan or laminarin resulted in inhibitions of 34.5% and 30.0%, respectively, while co-incubations with mannose yielded a 42.8% inhibition (Fig. 7B). Co-incubation of mannose plus curdlan led to an inhibition of 38.4%, whereas mannose plus laminarin displayed a 66.4% inhibition (*P* < 0.05, Fig. 7B). Overall, the more pronounced inhibitory effects observed in both phagocytes for the combination treatments corroborate the presence of distinct recognition systems for β-1,3-glucans and mannose, as expected.

### Fungal entrance through β-1,3-glucan recognition contributed to fungal survival within *A. castellanii*

Similar experimental setups were employed to investigate the interactions in the presence and combination of curdlan, laminarin, and mannose, and their impact on fungal survival within both phagocytes. Following an overnight incubation, the yeasts were recovered, and fungal viability was assessed. For *Ac*, co-incubations with curdlan or laminarin resulted in a reduction of 25.7% and 23.9% in the number of colony-forming units (CFUs) (Fig. 7C, *P* < 0.05), compared to the control in the absence of polysaccharides. However, co-incubation with mannose alone resulted in a 44.0% reduction (*P* < 0.0001) in the number of viable yeasts, indicating a mutual dependency of both recognition mechanisms for fungal entry, maintenance, and survival within amoebae. Furthermore, the combinations of curdlan or laminarin with mannose did not further impact fungal viability compared to individual treatments. Similarly, when RAW macrophages were used, co-incubations with curdlan or laminarin also affected fungal survival, resulting in a 25.4% and 34.3% reduction in fungal growth, respectively, compared to controls (Fig. 7D, *P* < 0.05). Co-incubation with mannose alone led to a 19.4% reduction in fungal survival. As observed for *Ac*, combinations of polysaccharides did not result in additional inhibition of fungal growth in macrophages. These results highlight the role of the distinct recognition systems for β-1,3-glucan and mannose in modulating fungal survival within phagocytes.

### Determination of proteins with affinity to β-1,3-glucans in the *A. castellanii* biotinylated surface extracts

**Fig 8:**
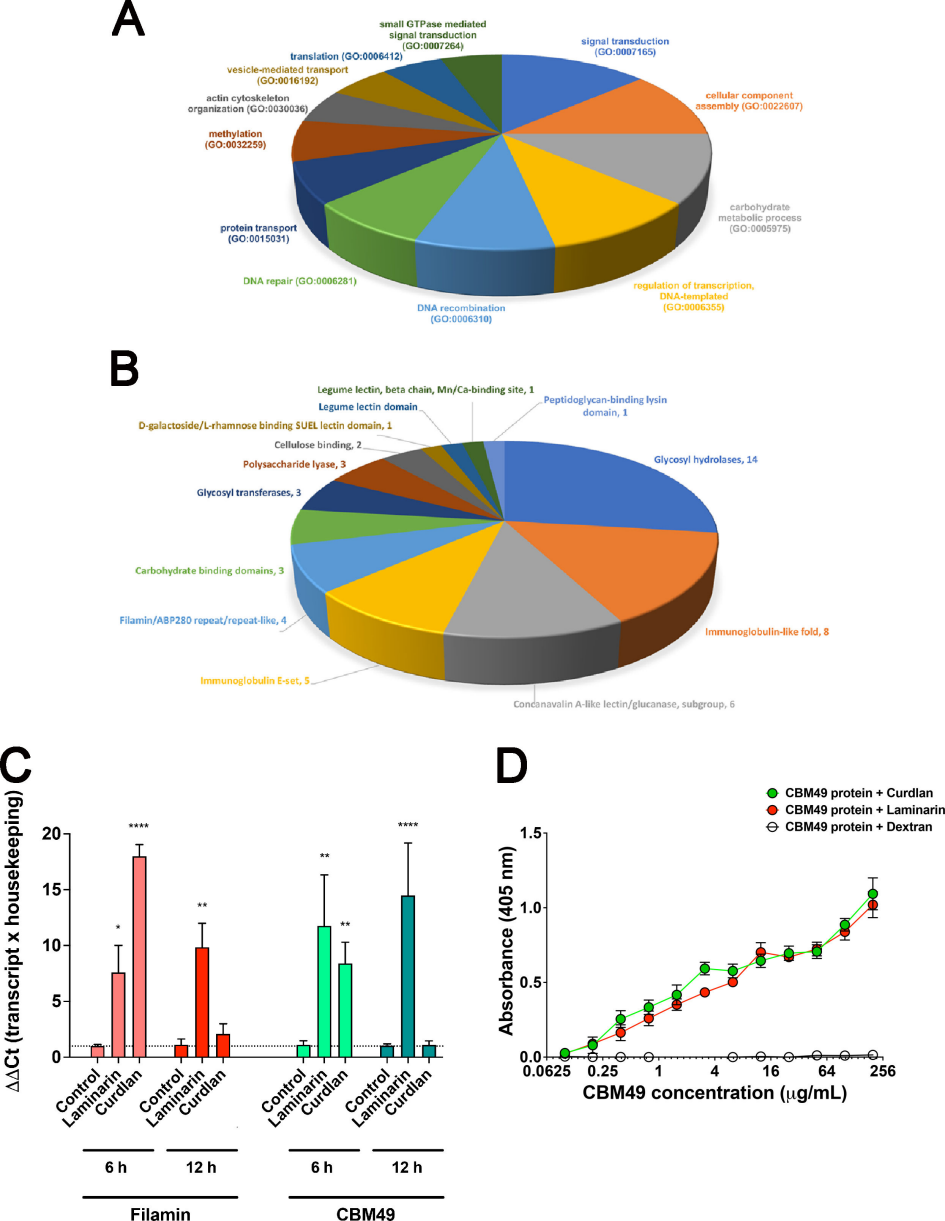
Enrichment analysis and characterization of proteins with affinity to β-1,3-glucan in *A. castellanii* identified by *de novo* sequencing using PEAKS X and PepExplorer. (A) From the proteins identified, 52 proteins with recognized affinity to β-1,3-glucan were classified into 12 main groups according to (A) Gene Ontology and (B) protein domains mapping using the InterPro database. From all the proteins identified, specifically the filamin (L8HDD6) displayed a legume lectin domain/ beta chain/ Mn/Ca_binding site (IPR019825) which overlapped with a ConA-like/glucanase domain homologous superfamily (IPR013320) domain, while the Carbohydrate binding CBM49 domain-containing protein (L8HAP9), which is a Luke lectin, displays two CBM2/CBM3 carbohydrate-binding domain superfamily (IPR008965) domains and three carbohydrate-binding CBM49 (IPR019028) domains. Due to their potential capacity to bind polysaccharides, both were followed up to 48 h regarding their expression levels during interactions with β-1,3-glucan and *H. capsulatum*. (C) Filamin (L8HDD6) and (D) CBM49 containing protein (L8HAP9) had their expressions increased upon incubations with β-1,3-glucan. (D) Indirect ELISA displaying the affinity of the purified CBM49 protein to curdlan (green line) and laminarin (red line).

Shotgun mass spectrometry data were submitted to *de novo* sequencing and the identified peptides were evaluated by the PepExplorer software. A vast list of identified peptides and matching proteins are displayed in Table S1, along with the number of unique peptides, alignment/spectra, molecular weight, Gene Ontology terms, and additional homologous superfamily mapped domains. The majority of proteins are involved in signal transduction (GO:0007165), followed by proteins involved in cellular component assembly (GO:0022607) and carbohydrate metabolic process (GO:0005975) (Fig. 8A). Additionally, proteins bearing domains involved in carbohydrate and polysaccharides recognition were heavily present, from glycosyl hydrolases to immunoglobulin-like fold, concanavalin A-like lectin/glucanase, filamin/ABP280 repeats, specific carbohydrate-binding domains, and glycosyl transferases (Fig. 8B). Despite the number of protein hits, two domains stood out for being among the highest average enrichment scores. The legume lectin domain, beta chain, Mn/Ca_binding site (IPR019825) represented by the single hit filamin repeat domain-containing protein (L8HDD6, ACA1_149410), overlapped with its ConA-like/glucanase domain homologous superfamily domain (IPR013320; 40-264 aa, Fig. S1A), which would confer the capacity to bind polysaccharides. Following, the carbohydrate binding domain CBM49 (IPR019028) was represented by two carbohydrate-binding domain CBM49 containing proteins (L8HAP9, ACA1_252830 and L8GUY9, ACA1_304510). The L8HAP9 is a cyst-wall Luke lectin containing a CBM2/CBM3, carbohydrate-binding domain superfamily (IPR008965), three CBM49 domains, and no transmembrane domain (Fig. S1B), which might be also involved in polysaccharide binding (33).

### Expression of selected proteins upon co-incubations with laminarin and curdlan

The transcription levels of the proteins bearing two of the enriched domains, the legume lectin domain, beta chain, Mn/Ca_binding site (IPR019825), and the carbohydrate-binding domain CBM49 (IPR019028), respectively, the L8HDD6 (ACA1_149410) and the L8HAP9 (ACA1_252830), were assessed by real-time PCR upon challenge of *Ac* with laminarin and curdlan, as described for 6 h, 12 h, and 48 h. As early as 6 h, the filamin transcript levels were increased upon contact with laminarin and curdlan (Fig. 8C). Only in co-incubations with laminarin, the levels of filamin transcripts were still elevated at 12 h and 48 h. For the CBM49 protein L8HAP9, co-incubations with laminarin and curdlan were able to increase the transcript levels upon 6 h, and as observed with filamin, only the co-incubations upon 12 h seemed to maintain the overexpression of this protein (Fig. 8C).

### CBM49 domain-containing protein binds to curdlan and laminarin

To validate our findings, we expressed and purified the CBM49 utilizing affinity chromatography. Upon induction of protein expression in the pET15b-CBM49 (Fig. S2A) transformed *Escherichia coli* with isopropyl-β-D-thiogalactopyranoside (IPTG) for 4 h, we observed an increase in the levels of a band of approximately 50 kDa, as detected by WB using anti-HisTag mAbs (Fig. S2B). As a negative control for expression, we also analyzed non-induced cell pellets, which showed no reactivity. The observed molecular weight aligns with the information regarding the CBM49 (L8HAP9) deposited in the UniProtKB database, indicating a protein of 423 amino acids and approximately 43 kDa. The purified recombinant CBM49 was assessed for its binding against β-1,3-glucans, laminarin, and curdlan using ELISA. We observed a dose-dependent reactivity, displaying similar binding affinities to both polysaccharides in comparison to the absence of reactivity seen with dextran controls (Fig. 8D).

## DISCUSSION

Amoebae are natural predators and adeptly exploit microorganisms in their milieu, particularly through phagocytosis of viruses, bacteria, and fungi, to obtain the nutrients indispensable for their biological activities and growth. Recognition of these microbes is often mediated by lectin-like transmembrane protein receptors, binding to target polysaccharides on the surface of the phagocytosed microorganisms (4, 5, 21).

The bacteria *Legionella pneumophila* is recognized by *Ac* through a lectin-like MBP, which selectively engages mannose moieties on the bacterial surface (19, 34, 35). Declerck et al. in 2007 described that upon the addition of soluble mannose during the *L. pneumophila-Ac* interaction, a dose-dependent inhibition of the uptake of bacteria by amoeba is observed (19). In this work, the authors also described for the first time a lectin-like receptor capable of recognizing N-acetyl-D-galactosamine on the surface of bacteria by *Naegleria lovaniensis*, a process which could also be inhibited by soluble monosaccharides.

Recently, our group elucidated at the molecular level the interactions between pathogenic yeasts and *Ac* (21) by demonstrating the critical roles of variables such as multiplicity of infection (MOI) and time, the extrusion-like phenomena (vomocytosis), and the inhibitory effect of soluble mannose during yeast-*Ac* interactions. The presence of *Ac* surface proteins able to bind to synthetic or fungal-associated mannose residues plays a crucial role during the fungal-*Ac* interaction. Fungal affinity precipitation, followed by magnetic beads protein purification and mass spectrometry, identified two proteins, MBP (L8GXW7) of 55 kDa and MBP1 (Q6J288) of 130 kDa, both biochemically characterized as fungal receptors and further validated upon molecular modeling *in silico* (21).

The most common post-translational modification on the fungal surface is the O-mannosyl glycans, which can result in different mannose monomer configurations. However, these do not constitute the most abundant carbohydrates on the fungal surface (36). The β-1,3-glucan is one of the most expressed polysaccharides on the fungal cell wall and is usually the preeminent dry-weight component of this structure (37). β-1,3-glucan is recognized by phagocytic cells of the immune system, such as macrophages, neutrophils, and dendritic cells (DCs), through a β-1,3-glucan-binding C-type lectin receptors (CLRs), called Dectin-1 (38), which triggers the initiation of the entire signaling cascade for the immune response against the pathogen (29).

To date, numerous reports have demonstrated the dependence on the β-1,3-glucan recognition for the interactions between microorganisms and phagocytic cells, such as the case of the protozoa *Leishmania major*, and macrophages (39, 40). In fact, β-1,3-glucan recognition appears to constitute a conserved mechanism prevalent among phagocytic cells. Invertebrates such as insects, devoid of acquired immunity, boast powerful mechanisms in response to the parasite invasion that rely on the insect's innate immune cell expression of β-1,3-glucan-binding proteins to recognize microbial surface polysaccharides (41). β-1,3-Glucan-binding proteins have been identified in various species (40, 42–46). Typically, these proteins showcase two distinct domains: a C-terminal glucanase-like domain exhibiting homology with glucanases like xylanase or starch hydrolyzing enzymes and an N-terminal β-1,3-glucan recognition domain (39, 41, 47).

Studies aimed at characterizing signaling via Dectin-1 are commonly conducted using curdlan, a bacterial-derived linear β-1,3-glucan, and laminarin, an algae-derived β-1-3-glucan containing β-1-6-glucan branches; both can play a role in both innate and adaptive immunity. The affinity with curdlan and laminarin was evaluated by Brown in 2001, where both were able to bind to Dectin-1. Remarkably, these polysaccharides reduced by 90% and 85%, respectively, the attachment of zymosan to the surface of NIH3T3 cells (38).

Within macrophages, CLRs such as Dectin-1, Dectin-2, and some collectins, assume a pivotal role in the immunity against *Hc*. Dectin-1 and Dectin-2 significantly contribute to the development of Th1 and Th17 cells, which in turn orchestrate the antifungal response (48, 49). In synergy, CR3 and Dectin-1 collaborate to elicit the expression of tumor necrosis factor (TNF) and interleukin-6 (IL-6) by murine macrophages through a Syk-JNK-AP-1-dependent mechanism (50). While CR3 participates in the phagocytosis and cytokine responses across various cells, Dectin-1 engages and participates in the production of cytokines only in murine macrophages (45, 51). Pathogenic *H. capsulatum* yeasts secrete Eng1, a β-glucanase that hydrolyzes β-1,3-glucan glycosylic bonds, thus diminishing the yeast surface exposure of β-1,3-glucans, thus allowing *H. capsulatum* to evade detection by Dectin-1. *Hc* yeasts deficient for Eng1 manifest an increased binding to Dectin-1 and an enhanced production of TNF-α and IL-6 in murine macrophages and DCs (45, 52–54).

*Acanthamoeba* and macrophages ingest microbes via phagocytosis, an actin-dependent process culminating in pseudopodia formation (26, 55). Both macrophages and *Acanthamoeba* also share similarities at molecular and structural levels, biochemical regulation, and cellular motility, highlighting a possible evolutionary relationship (24, 26, 56). Overall, much of the understanding about the cytoskeleton, cell motility, involvement with pseudopodia, invagination, phagolysosome formation, membrane recycling, and the underlying mechanisms come from studies on *Acanthamoeba* (24, 26).

As in macrophages, the proteins present in the *Ac* membrane extracts bound to curdlan and laminarin. Thus, the identification of β-1,3-glucan-binding proteins became feasible. As expected for macrophages, curdlan has a discrete higher binding, attributed to its role as a Dectin-1 agonist (57, 58), as opposed to the Dectin-1 antagonist functions of laminarin (38, 59). In trophozoites, both polysaccharides displayed similar binding to *Ac* surface proteins, which could point out to distinct mechanisms of β-1,3-glucan recognition than macrophages.

Laminarin has been extensively studied and primarily employed in recent years due to its interaction with the glucan-specific pattern recognition receptor (PRR) (60), Dectin-1, to understand the importance of this molecule on fungi interactions (29, 45, 47). As an example, Brown et al. (29) reported that the CLR Dectin-1 is the major receptor responsible for the binding of fungal β-glucans and eliciting innate immune responses by phagocytic cells of the immune system (29, 45, 47).

Based on this idea, we selected curdlan and laminarin as the polysaccharide of choice for purifying the membrane extracts of *Ac* and other mammalian cell lines (macrophage RAW and CHO) (28, 61). Through an indirect ELISA, we assessed the capacity of *Ac*, RAW, and CHO biotinylated surface membrane extracts to bind curdlan and laminarin. Notably, amoeba extracts displayed comparable affinities for both polysaccharides. In contrast, RAW macrophage extracts displayed a higher affinity to laminarin, potentially attributed to the constitutive expression of β-1,3-glucan receptors on their surfaces (45). As expected, the absence of β-1,3-glucan receptors on the surface of CHO cells resulted in a lack of reactivity. In fact, stripping β-1,3-glucan-coated plates upon purification of the biotinylated surface proteins of *Ac* revealed numerous amoeba proteins with potential binding capabilities to β-1,3-glucan.

Regarding the MBPs of *A. castellanii,* it was conclusively demonstrated that their high affinity binding to mannose was in a dose/time-dependent manner, and such possibility should also be extended to potential *Ac* proteins binding to curdlan and laminarin. To investigate this hypothesis, we performed an optical tweezers-based adhesion assay using curdlan- or laminarin-coated polystyrene beads and we showed that both bound with high affinity to *Ac* in comparison to controls. The specificity of the interaction, i.e., the strict dependence on β-1,3-glucan recognition, was assessed using two distinct strains of *H. capsulatum*, *Hc* G217B and *Hc* G186A. *Hc* G217B has a freely exposed surface β-1,3-glucan, whereas the *Hc* G186A expresses an outer layer of α-1,3-glucan which hinders the recognition of β-1,3-glucan by mammalian phagocytic cells (31, 54, 62). *Ac* trophozoite bound strongly to *Hc* G217B, but this binding was drastically reduced during co-incubations with soluble laminarin; in contrast, the *Hc* G186A strain displayed very low attachment to *Ac* trophozoites, due to the limited accessibility of β-1,3-glucan caused by a steric hindrance imposed by the α-1,3-glucan layer. These observations were confirmed using an inhibition ELISA, which displayed more inhibition of the *Hc* G217B strain than *Hc* G186A for the binding of β-1,3-glucan purified biotinylated surface proteins to immobilized laminarin. By fluorescence, *Hc* G217B labeling with β-1,3-glucan purified biotinylated surface proteins displayed a ring-like pattern of labeling around the entire fungal wall, which co-localized with Dectin-1-Fc and Uvitex 2B. That labeling is consistent, given the continuous disposition of β-1,3-glucan throughout the fungal cell wall structure, despite the ongoing remodeling of the cell wall and dynamic distribution of those polysaccharides (31, 37).

The association rate of *Hc* G186A to *Ac* was 50% lower when compared to *Hc* G217, suggesting the overall importance of β-1,3-glucan recognition during the *Hc-Ac* interaction. A similar behavior was observed with RAW 264.7 macrophages. For both cells, co-incubations with either laminarin or curdlan during interaction with *Hc* G217B displayed a similar inhibition in the association rates. However, for both *Ac* and RAW 264.7, combinations of mannose plus β-1,3-glucans seem to have an addictive inhibitory effect, indicating the participation of overlapping pools of receptors for the interaction with both polysaccharides, i.e., these phagocytes possess a second mechanism that can bind fungi using a strategy as alternative to MBPs.

Blockage of these polysaccharide-recognizing pathways resulted in lower growth of intracellular *Hc* G217B for both *Ac* and RAW 264.7. Inhibition with mannose was accompanied by a reduction in the number of CFUs. As initially suggested by our group and further explored in recent studies, the MBPs are lectins involved in the recognition of mannose residues on the surface of pathogenic yeasts (21, 28). Impairment in mannose recognition results in lower fungal entrance and survival within *Ac* and macrophages, suggesting the pivotal role of these pathways in fungal adaptation and survival within these phagocytes (28). In fact, mannose recognition seems to be a complex process involving the participation of numerous proteins in both models, including carbohydrate and polysaccharide binding proteins, glycosyl hydrolases, and transferases. *Ac* displays a simpler protein repertory for mannose recognition and a low number of analog genes, which are probably involved in nutritional purposes and for the survival of amoeboid organisms. In comparison to macrophages, these genes could have undergone paralogical genetic evolution from ancestor genes, generating more complex mannose recognition pathways in higher organisms, as seen in innate immune cells, such as macrophages.

Combinations of curdlan or laminarin with mannose demonstrated the same growth inhibitory effect as the curdlan or laminarin alone, which was lower than the sole mannose inhibition. These again might indicate the participation of overlapping proteins in the recognition of both molecules, which needs to be further confirmed. However, the exact profile of proteins involved in fungal recognition might change in both scenarios, abrogating the pronounced inhibitory effect of mannose alone. Therefore, the association of mannose and β-1,3-glucan recognition pathways might allow fungal entrance to phagocytes and better fungal adaptation to the intracellular milieu of these phagocytic cells. However, the killing mechanism used by *A. castellanii* needs to be fully elucidated.

The shotgun proteomics identified a long list of proteins with the capacity to bind to β-1,3-glucan. However, one of the limitations of this elected methodology is that it does not rule out the co-purification of proteins that have no direct binding to β-1,3-glucan, being part of protein complexes. Gene Ontology mapping displayed most proteins involved in biological processes such as signal transduction (GO:0007165), cellular component assembly (GO:0022607), carbohydrate metabolic processes (GO:0005875), and actin cytoskeleton organization (GO:0030036), which, among them, displayed the highest enrichment. Cazy, a repository for carbohydrate-active enzymes (www.cazy.org) for protein mapping, includes classes of glycoside hydrolases and transferases, polysaccharide lyases, carbohydrate esterases, and proteins bearing carbohydrate modules. Following their recommendations, we found enriched domains in our list, including glycosyl hydrolases, immunoglobulin-like fold, concanavalin A-like lectin/glucanase, filamin/ABP280 repeats, and specific carbohydrate-binding domains (Fig. 8B). From these, two proteins also mapped to GO molecular functions of carbohydrate (GO:0030246) and polysaccharide binding (GO:0030247), which were, respectively a filamin (L8HDD6) and a CBM49 domain-containing protein (L8HAP9). Both proteins were followed regarding the expression levels during incubations with laminarin, curdlan, and *Hc*, displaying higher levels of expression.

In particular, one protein identified in our analysis, a filamin repeat domain-containing protein (L8HDD6), was also specifically classified as a legume lectin displaying an overlapping domain to ConA-like/glucanase domain superfamily. This protein contained an important transmembrane domain similar to the epidermal growth factor receptor family of protein tyrosine kinases and could fit the role of a biosensor/adaptor for the β-1,3-glucan recognition (34, 59, 60). Additionally, the CBM49 domain-containing protein (L8HAP9) has been demonstrated to bind to polysaccharides on the surface of trophozoites during encystation (33). As this protein also appears in our analysis, its binding capacity was validated upon expression in the prokaryotic system and demonstrated a high affinity to both curdlan and laminarin. We cannot rule out the participation of any protein identified by mass spectrometry as a potential PRR, adaptor or part of a protein complex for β-1,3-glucan recognition. Therefore, due to the molecular complexity encountered, additional studies are yet to be performed using genetic manipulation of trophozoites and mutant construction to solidly identify any receptor involved in the β-1,3-glucan attachment aiming the fungal recognition and subsequent phagocytosis.

*Ac* resembles human macrophages in many ways, including morphological, molecular, biochemical, and functional levels, particularly in its cell surface binding to pathogens (16, 19, 21, 23, 28, 50, 61). The β-1,3-glucan recognition seems to be an important pathway shared by these two phagocytes and the similarities and molecular mimicry on the recognition of these polysaccharides and fitness between these distantly related species need to be explored, including the participation of receptors in the pathogen recognition process of both cells, even to establish a possible even closer relationship between *Ac* and macrophages.

## MATERIALS AND METHODS

### Microorganisms and growth conditions

*Ac* ATCC 30234 was obtained from the American Typing Culture Collection (ATCC, Manassas, VA) and grown in a PYG medium [20 g/L peptone, 1 g/L yeast extract and 18 g/L glucose (pH 6.5) supplemented with 0.4 mM CaCl_2_, 0.4 mM MgSO_4_, 2.5 mM Na_2_HPO_4_, 2.5 mM KH_2_PO_4_, 1 g/L sodium citrate and 0.05 mM Fe(NH_4_)_2_(SO_4_)_2_]. *Ac* was maintained in cell culture flasks at 28°C until confluence and used in the subsequent experiments as described (8). *Hc* G217B (ATCC 26032) or G186A (ATCC 26029) were cultured in HAMF-12 at 37°C for 48 h under shaking at 180 rpm (63).

### Mammalian cell culture

RAW 264.7 macrophage (ATCC TIB-71) and CHO cells were obtained from the Cell Bank of Rio de Janeiro, Brazil. Both cells were cultivated in a Dulbecco's modified Eagle medium (DMEM) supplemented with 1.2  g/L NaHCO_3_, 5% fetal bovine serum (Cultilab, Brazil), 2% non-essential amino acids (Thermo Scientific), and 1% penicillin/streptomycin (Thermo Fisher Scientific, Waltham, MA, USA) at 37°C with 5% CO_2_.

### Detection of β-1,3-glucan recognition on the surface of phagocytic cells *A. castellanii* and macrophages

RAW macrophages and *Ac* trophozoites were suspended at a density of 10^6^ cells/mL. Five different groups were established for each phagocyte: unlabeled controls, primary and secondary antibodies controls, and systems pre-incubated with 10 µg/mL of different polysaccharides (curdlan or laminarin; Merck Millipore, Burlington, MA, USA). For the experimental groups, cells were pre-incubated with sterile phosphate buffer saline (PBS; 140 mM NaCl, 2.7 mM KCl, 1.5 mM KH_2_PO_4_, 17 mM Na_2_HPO_4_, pH 7.2) (Merck Millipore) or specific polysaccharides for 45 min at room temperature (RT). Subsequently, all the systems were washed three times with PBS by centrifugation at 800 g for 10 min and resuspension. The cells were fixed with 4% paraformaldehyde for 30 min at 37°C, followed by three washes with sterile PBS. Next, cells were blocked with 1% BSA in PBS for 1 h at RT. After the blocking step, the cells were again washed three times with sterile PBS and incubated with Dectin-1-Fc (mouse IgG2a) primary antibody at a concentration of 5 µg/mL for 1 h at RT, as previously described (46). The anti-mouse IgG-Alexa 488 was added as the secondary antibody for 1 h at RT, and the systems were washed again three times. The primary and secondary antibodies were performed independently for both phagocytes as controls for autofluorescence and non-specific reactivity, respectively. All samples were submitted to flow cytometry analysis using the BD FACSCalibur (BD Biosciences, San Jose, CA, USA) to detect the FL1 fluorescence intensity of the cells. The acquired data were analyzed using the FlowJo X software (Becton, Dickinson & Company, Franklin Lakes, NJ, USA).

### Biotinylation of mammalian cells and *Acanthamoeba castellanii* surface proteins

Surface proteins of mammalian cells (CHO and macrophages) and trophozoites of *Ac* were biotinylated after 48 h of culturing under ideal conditions, where the cells reached confluence and viability of ≈100%. Cells were washed three times with PBS (pH 8.0) at 4°C to remove amine and protein residues from the culture media, and each wash was followed by centrifugation at 220 × *g* for 10 min. Subsequently, 25 × 10^6^ cells were incubated with Sulfo-NHS-LC-Biotin (Thermo Fisher) at a final concentration of 2 mM for 30 min at 4°C, homogenizing every 5 min. After 30 min, three washes were performed with a cold PBS (pH 8.0) containing 100 mM glycine to neutralize the excess biotin. The cells were suspended in an extraction buffer (0.5% CHAPS; 2M β-mercaptoethanol; 25M Tris-HCl; 100M NaCl; 20M CaCl_2_; 1 mM phenylmethylsulfonyl fluoride) at 4°C, with additional protease and phosphatase inhibitors [Halt Protease and Phosphatase Inhibitor Cocktail, EDTA-free (100×), Thermo Fisher], and then the samples were homogenized at 4°C using 20 cycles in a Dounce homogenizer. Following the homogenization step, the cells were centrifuged at 18,000 g for 30 min at 4°C, and the supernatant was collected. Protein quantification was performed using the Bicinchoninic acid (BCA) kit (Thermo Fisher). The samples were incubated with sample buffer (4×) (Thermo Fisher) and protein separation was carried out using a 12% SDS-PAGE gel using the NuPage system (Thermo Fisher) and MOPS-SDS running buffer (Thermo Fisher) at 100V voltage/constant amperage of 40 mA. The SDS-PAGE gel was stained using the Pierce Silver Stain Kit (Thermo Fisher), following the manufacturer’s protocol.

### ELISA against distinct presentations of β-1,3-glucans

A 96-well ELISA microplate was coated using 10 µg/mL of curdlan and laminarin and then incubated overnight at 4°C. Following incubation, the wells were thoroughly washed three times with a 0.1% Tris base saline-Tween buffer (TBS-T, 10 mM Tris, 150 mM NaCl, and 0.1% Tween 20; pH 7.4). Subsequently, the wells were blocked with 300 µL of Superblock (Thermo Fisher) for 1 h at RT. Biotinylated surface extracts of phagocytes or CHO control extracts were diluted in Superblock at concentrations ranging from 150 to 3.13 μg/mL, and added to plates (50 µL/well), followed by incubation for 1 h at 37°C. Afterward, the plates were washed three times with TBS-T and a 1:2,000 dilution of streptavidin-alkaline phosphatase conjugate (Thermo Fisher) was added to each well, followed by a Superblock and an additional 1 h incubation at 37°C. Finally, the plates were washed three times and the ELISA was developed using the p-nitrophenyl phosphate (pNPP) substrate (Merck Millipore) as per the manufacturer’s instructions. The absorbance at 405 nm was measured at RT using a SpectraMax microplate reader M2e (Molecular Devices, San Jose, California, USA).

### Isolation of β-1,3-glucan-binding protein extracts

To isolate the β-1,3-glucan-binding proteins, a 48-well polystyrene plate was coated overnight with either curdlan or laminarin, both diluted in PBS and incubated at 4°C. Subsequently, the plate was washed with divalent cation PBS (DPBS; PBS pH 7.5 plus 0.49 mM MgCl_2_ and 0.90 mM CaCl_2_) to remove the excess polysaccharides. Then, the biotinylated surface extract of *Ac* was diluted in DPBS to a final concentration of 100 µg/mL and added to the plate, which was incubated overnight at 4°C. Afterward, the wells were washed, and the proteins bound to the adhered curdlan or laminarin were extracted using a stripping buffer [50 mM Tris-HCl, 2% SDS, 50 mM dithiothreitol (DTT), pH 7]. Effective stripping with this buffer usually required incubation for 30 min at 70°C, and some denaturation and loss of the target protein were unavoidable. As this buffer contains DTT, which is unstable, it must be prepared immediately before use (56). The obtained proteins were collected and dialyzed in Slide-A-Lyzer Dialysis Cassettes, 10K MWCO, 3 mL (Thermo Fisher) against DPBS (pH 8.0) for 48 h at RT with three buffer exchanges. The samples were then collected and quantified using the BCA kit (Thermo Fisher). The recovered proteins were evaluated by SDS-PAGE, as described above. For WB, the proteins present on the gel were transferred to a polyvinylidene difluoride (PVDF) membrane which was pretreated with 100% methanol for 5 min and washed for another 5 min with the transfer buffer (3 g/L Tris base, 14.4 g/L glycine, and 20% methanol). The transfer was performed using a voltage of 160 V and 300 mA current for 1 h at 4°C in transfer buffer. Subsequently, the membranes were washed three times with TBS-T 0.1% (TBS and at 0.1% Tween 20) for 5 min each and blocked with 5% skim milk in TBS-T for 1 h with gentle shaking. After three additional washes, a streptavidin-horseradish peroxidase conjugate (Merck Millipore), diluted 1:10,000 in blocking buffer, was added and incubated for 1 h at RT. The membrane was washed three times with TBS-T for 10 min per wash, developed using the SuperSignal West Pico PLUS Chemiluminescent Substrate (Thermo Fisher), and imaged using the Gel Doc XR + Gel Documentation System.

### Optical tweezers

Polystyrene beads (3 µm diameter, Polysciences, PA, USA) were coated with 10 µg/mL of either curdlan or laminarin overnight at RT. After three washes with PBS and centrifugation at 1,100 × *g*, the beads were enumerated using a hemocytometer. Beads coated with either 1% BSA or 10 µg/mL dextran (containing a high concentration of α-1,4-glucan polysaccharide) served as negative controls (Merck Millipore). *Ac* (10^4^ cells) in PYG were plated on a Nunc Glass bottom dish (Thermo Fisher) and allowed to incubate for 1 h to promote cell adhesion. Polysaccharide-coated beads were added to the dishes containing *Ac* in a 1:1 bead:amoeba ratio. Optical tweezers were employed to measure the adhesion rates of the polysaccharide-coated beads to the surface of *Ac*, following previously described methods (51, 64). Beads in suspension were captured by the optical tweezers laser and brought into contact with the surface of *Ac*, for periods ranging from 5 s to 90 s. Subsequently, the microscope stage was moved in the opposite direction to detach the laser-trapped beads from the *Ac* trophozoite. If detachment was achieved, it was considered a positive adhesion event. The relative adhesion or the ratio of positive adhesion events NN0 , was measured for each time interval $\left(t\right)$ of contact. The characteristic time (τ, representing the required time for 63% of the adhesion events to be positive) was calculated for each experimental condition based on the best fit for all the obtained curves, according to the following equation:


(1) $$\frac{N}{N_{0}}=1-exp\left(-\frac{t}{\tau }\right)$$

The error bars for each time point were determined as half the difference between the maximum and minimum values in 30 events performed with three different samples. The errors in τ values were obtained using the best curve fit for Equation (1) and also weighing the data with the errors for the relative adhesions and compared.

Additionally, two distinct strains of *H. capsulatum*, namely *Hc* G217B (ATCC 26032) and *Hc* G186A (ATCC 260290), were used to confirm the involvement of β-1,3-glucan on the *Ac*-fungi adhesion. *Hc* G217B expresses the β-1,3-glucan, while *Hc* G186A expresses an additional external layer of α-1,3-glucan. Experiments were conducted as described above, and characteristic times for the adhesion of both yeasts were determined and compared. To further validate the importance and specificity of β-1,3-glucan in the amoeba-*Hc* interaction, experiments were performed in the presence of 10 µg/mL laminarin. All plots and curve fit were obtained using the Kaleidagraph software (Synergy Software, Essex Junction, VT, USA).

### Binding evaluation of *A. castellanii* biotinylated surface β-1,3-glucan-binding proteins to fungi by immunofluorescence and flow cytometry

To further characterize the binding of *Ac* β-1,3-glucan-binding proteins to fungi, we employed fluorescence microscopy and flow cytometry techniques following established protocols (64, 65). *Hc* yeasts were cultured and subsequently washed three times with PBS (by centrifugation at 1,100 g for 10 min each) before being fixed with 4% paraformaldehyde for 1 h. After fixation, the yeasts underwent an additional three washes and were blocked with 2% BSA in PBS. Next, the *Ac* biotinylated surface β-1,3-glucan-binding proteins (100 µg) were diluted in 1 mL of 2% BSA in DPBS and incubated with 10^7^ yeasts for 2 h at RT with gentle shaking. Following the incubation, the yeasts were washed three times with DPBS and then centrifuged. Subsequently, the cells were incubated with a streptavidin‐Alexa 488 conjugate (SouthernBiotech) diluted to 5 µg/mL with 2% BSA-DPBS for 1 h at RT. As a control for the presence of β-1,3-glucan on the fungal surface, the yeasts were incubated with 5 µg/mL Dectin-1-Fc (32, 46) in 2% BSA-DPBS for 1 h at RT. After three washes with DPBS, yeasts were further incubated with an anti-mouse IgG Alexa 546 conjugate (SouthernBiotech) diluted to 5 µg/mL with 2% BSA-DPBS for 1 h at RT. Lastly, the yeasts were incubated with 0.5 mg/mL Uvitex 2B (Polysciences, USA) for 30 min, followed by three additional washes with DPBS. Subsequently, the yeasts were examined under an AxioImager microscope (Carl Zeiss MicroImaging Inc., USA) at 100×. Additionally, the preparations were analyzed using a FACSCalibur flow cytometer (BD Biosciences, USA), and the fluorescence intensity of FL1+ cells (for *Ac* biotinylated β-1,3-glucan-binding proteins + streptavidin‐Alexa 488) or FL2+ (for Dectin-1-Fc + anti-mouse IgG Alexa 546) was compared with controls in the absence of clarified *Ac* biotinylated surface proteins extract (or fluorophore conjugate controls) (21, 28).

### Binding evaluation of *A. castellanii* biotinylated surface β-1,3-glucan-binding proteins to fungi by ELISA

The binding of *Ac* biotinylated surface β-1,3-glucan-binding protein to fungi was evaluated by an inhibition ELISA. Briefly, a microplate (reaction plate) was coated with 50 µL of a 10 µg/mL solution of laminarin for 1 h incubation at 37°C, followed by an overnight incubation at 4°C. The reaction plate, along with a second plate (inhibition plate), was blocked with 1% BSA in DPBS for 1 h at 37°C. After blocking, fungal cells (ranging from 10^7^ to 10^2^ per well) were added to the inhibition plate and incubated for 1 h at 37°C with 50 µg/mL of *Ac* biotinylated surface β-glucan-binding proteins, which were diluted in the blocking buffer. Subsequently, the content of the inhibition plate was completely transferred to the blocked reaction plate, and the plates were further incubated for 1 h at 37°C. After three washes with DPBS, plates were added of 1 µg/mL of streptavidin-alkaline phosphatase (Thermo Fisher) in blocking buffer and incubated for 1 h at 37°C. Then, plates were washed three times and developed using pNPP. The absorbance values were recorded by measuring the plates at 405 nm.

### Interaction of *H. capsulatum* with trophozoites and macrophages

*Ac* trophozoites or RAW macrophages were plated in a cell culture plate (24 wells) at 5 × 10^5^ cells/well in PYG or DMEM medium as aforementioned. The cells were then incubated with 100 µM of either curdlan, laminarin, mannose, or their combinations diluted in PYG or DMEM, respectively, for 1 h at 28°C or 37°C*. H. capsulatum* yeasts were labeled with 40 µg/mL of NHS-Rhodamine (Thermo Fisher) for 1 h at RT. After the incubation, the cells were washed three times with excess sterile PBS, suspended in PYG or DMEM, enumerated, and added to the amoeba or macrophages at an MOI of 2 yeast:1 amoeba or macrophage. The plates were then incubated for 2 h at specific temperatures for each phagocyte. After three washes with PBS, the cells were detached from the wells by pipetting up and down and were fixed with a 4% formaldehyde solution in PBS. The interactions between amoebae/macrophages and yeasts were measured by flow cytometry using an LSRII BD (BD Biosciences) and at least 10,000 events were recorded. The data were subsequently analyzed using the FlowJo X software, and the association rates were determined as the ratio of phagocytes with interacting yeast cells (FL2+) divided by the total number of phagocytes measured (28, 51, 65).

### Yeast killing assay

After cultivation, *Hc* yeasts were washed as described above, and then they were diluted in PYG or DMEM and enumerated. Subsequently, yeasts were added to amoeba or macrophages at an MOI of 2 yeast:1 amoeba or macrophage, and interactions were allowed to proceed for 2 h of incubation in both systems as described (21). The plates were then washed to remove all yeasts that had not interacted with phagocytes, followed by an overnight incubation at 28°C for amoeba and 37°C under 5% CO_2_ for macrophages. The wells were then washed with cold PBS and amoebae/macrophages were lysed by adding sterile water. For amoebae, an additional step of passing them 10 times through a 26G1/2 syringe was performed to achieve optimal lysis. Aliquots were then plated on brain heart infusion (BHI)-blood agar plates containing 10 g/L glucose, 0.1 g/L cysteine, 1% penicillin/streptomycin, and 5% vol/vol sheep red blood cells. The plates were incubated at 37°C for 10–15 days, to allow for the enumeration of CFUs, which were subsequently compared among experimental groups (66).

### Sample preparation and identification of *A. castellanii* surface proteins with β-1,3-glucan affinity by mass spectrometry

One hundred micrograms of the aforementioned extract, in two biological replicates (and two experimental replicates), containing *Ac* biotinylated surface proteins with β-1,3-glucan affinity was incubated with 100 µg of streptavidin-coupled Dynabeads (Thermo Fisher) for 30 min at RT with shaking. The beads were washed five times with PBS using magnetic capturing and dried in a speed vac (Eppendorf, Germany) (21). The magnetic beads were suspended in a solution containing 7 M urea and 2 M thiourea and added of 1 M HEPES to a final concentration of 100 mM in solution. This mixture was further added 100 mM dithiothreitol to a final concentration of 10 mM and incubated for 1 h at 30°C. Alkylation was performed by adding 400 mM iodoacetamide to a final concentration of 40 mM, and the tubes were incubated at RT in the dark for 30 min. Afterward, 175 µL of ultrapure water (TEDIA, USA) and 100 ng/mL sequencing grade trypsin (Sigma-Aldrich) diluted in 0.1 M of acetic acid were added to a final concentration of 0.1 ng/mL. The tubes were then incubated overnight at 37°C. Subsequently, 2 µL of 10% trifluoroacetic acid (TFA) was added to the samples, which were cleaned up using a micro-Spin Column (Harvard Apparatus) and dried in a speed vac. The tryptic peptides were dissolved in 10 µL of 0.1% TFA and approximately 4 µL of the mixture was loaded onto an in‐house packed column (15 cm × 75 µm) filled with 3 µm ReproSil C-18 resin (Dr. Maisch GmbH), using the NanoLC-Ultra nano liquid chromatography system (Eksigent Technologies) (8).

### Shotgun mass spectrometry and data analysis

Samples were analyzed in a nano-LC-MS/MS system comprising an EASY II-nano LC system (Proxeon Biosystem, Denmark) coupled to a nanoESI LTQ-Orbitrap Velos mass spectrometer (Thermo Fisher). Two micrograms of peptides were loaded onto a trap column (100 µm × 2 cm) packed in-house with C-18 ReproSil 5 µm resin (Dr. Maisch) and a New Objective PicoFrit analytical column (75 µm × 20 cm) packed with Reprosil-pur C18-AQ 3 µm resin (Dr. Maisch). A linear gradient of a solvent solution consisting of 95% acetonitrile and 0.1% TFA was used for peptide elution, with the gradient ranging from 5% to 20% for 85 min, 20%–40% for 22 min, 40%–95% for 5 min, and 95% for 8 min, at a flow rate of 250 nL/min. Mass spectra were acquired in a positive mode top 10 data-dependent acquisition method. The MS1 scan was acquired in an Orbitrap analyzer set for a 350–1,800 m/z range, 60,000 resolution (at m/z 400) with a minimal signal required of 10,000, and isolation width of 2.0. The 10 most intense ions were subjected to fragmentation by collision-induced dissociation at 30 normalized collision energy, with a dynamic exclusion of 30 s. The acquired spectra were processed using the peptides search engine BSI PEAKS X (Bioinformatics Solutions Inc.) for peptide identification. The parent mass error tolerance and fragment mass error tolerance were both set to 0.6 Da and 0.5 Da, with precursor mass search type monoisotopic. The maximum allowed missed tryptic cleavages was set to two, and one non‐specific cleavage was permitted. Carbamidomethylation (molecular weight (MW) = 57.02 Da) was set as a fixed modification, while methionine oxidation (MW = 15.99 Da) and lysine sulfo-NHS-LC-Biotin conjugation (MW = 339.16 Da) were considered as variable modifications. The *Ac* database used was downloaded from www.uniprot.org and contained 14,944 proteins (as of February 2021). The considered false discovery rate was set to 1%. All processed data and spectra generated by the software were compared with raw data spectra and manually checked.

### *De novo* sequencing analysis

The Peaks X software generated a *de novo* peptide output, which was further analyzed using the *de novo* sequencing tool (PepExplorer) of the PatternLab for Proteomics platform (http://www.patternlabforproteomics.org). For this analysis, the same target-decoy sequence database of *Ac* as described previously was utilized, following instructions provided elsewhere (66). The parameters were carefully adjusted to optimize the analysis process and most stringent comparison criteria, including the Reverse Decoy Label insertion, a minimal identity threshold of 0.5, a minimum peptide size of six amino acids, and a *de novo* score cut-off of 90. Upon analysis, the dynamic report provided a list of identified proteins, along with their corresponding matching peptides based on sequence alignments. The results were compared among samples for the determination of proteins with affinity to β-1,3-glucan affinity. Only protein hits that exhibited a minimum of two unique peptides identified, with at least five amino acid residues sequenced consecutively in the series Y- or B- (or by complementary form) and with at least 5.0% coverage, were considered valid in the analysis (67–70). Protein annotation, gene ontology, domain identification, and enrichment analyses were performed using the DAVID (Database for Annotation, Visualization and Integrated Discovery; https://david.ncifcrf.gov/home.jsp) (71) and validated using the InterPro database (https://www.ebi.ac.uk/interpro/). Identified main protein classes and functional domains with recognized affinity to β-1,3-glucan were classified according to Legentil et al. (39) and the carbohydrate-active enzymes (www.cazy.org) repository. Besides, protein transmembrane domains were mapped using the Transmembrane Hidden Markov Model (TMHMM server v. 2.0; http://www.cbs.dtu.dk/services/TMHMM/) and the PSI-blast-based secondary structure PREDiction (PSIPRED 4.0; http://bioinf.cs.ucl.ac.uk/psipred) (17, 21, 72, 73).

### Quantitative Real-time PCR

*Ac* trophozoites were plated on 24-well plates and infected with an MOI of 2:1 of *Hc* G217B, and interactions proceeded for 2 h of incubations as described (21). The plates were washed to remove non-interacting yeasts. Additional groups consisted of *Ac* incubated with 100 µM of either curdlan or laminarin or control in the absence of polysaccharides. Plates were incubated for 6 h, 12 h, and 48 h at 28°C. Trophozoites were recovered by pipetting up and down and harvested by centrifugation at 800 *× g* for 10 min. After three washes with cold PBS, RNA was isolated using TōTALLY RNA Kit (Life Technologies) following the manufacturer’s instructions. RNA was further treated with DNAse, and concentrations were determined in a NanoDrop spectrophotometer (NanoDrop Technologies). RNA purity was confirmed by a ratio of absorbances 260/280 >1.8, and integrity was evaluated further by electrophoresis. For the cDNA, 1 µg of RNA was used and synthesis was performed using the ImProm-II Reverse Transcription System (Promega, WI, USA). The RT-qPCR reactions were performed in a thermocycler Stratagene Mx3005P using the Platinum SYBR Green qPCR SuperMix-UDG (Thermo Fisher) in 96-well white-opaque PCR plates sealed with adhesive films for qPCR. The qPCR mixer consisted of 15 µL Syber Green Mix, 10 µM of each primer (Table S2), and 50 ng cDNA (5 µL of 10 ng/µL). The 18SQV (18S rRNA gene) was elected as housekeeping for normalizations as described (74, 75). Negative controls were performed with no addition of cDNA templates. The qPCR consisted of an initial UDG incubation step at 50°C/2 min, followed by a denaturation step at 95°C at 2 min, and then 40 cycles of 15 s at 95°C and 1 min at 60°C. At the end of the RT-qPCR, melting curves were performed for each sample by increasing the temperature at a rate of 0.1°C/s from 65 to 95°C. Experiments were carried out in triplicate, each one with two experimental replicates.

### CBM49 expression and binding characterization

The coding sequence of CBM49 (L9HAP9) was optimized, synthesized, and inserted into the pET15b plasmid for *E. coli* expression (pET15b-CBM49opt-SfiI-EcoRI, Fig. S2A). *E. coli* (TOP10 strain, Thermo Fisher) was transformed with the pET15b-CBM49-SfiI-EcoRI plasmid and selected based on the resistance to ampicillin (100 µg/mL). Selected colonies were screened based on restriction enzyme profiles (*SfiI* and *EcoRI* used for cloning) and confirmed by sequencing. *E coli* was grown in Luria Bertani (LB) medium with ampicillin, until reaching an OD of 0.6, after which, 0.1 M IPTG was added for a 4-h induction of expression. Induced and non-induced control samples were collected and resolved by 10% SDS-PAGE. The efficiency of protein expression was confirmed by WB to 6xHisTag detection. Briefly, the samples were transferred to a nitrocellulose membrane, washed three times with TBS‐T for 5 min, and blocked with blocking buffer (5% skim milk in TBS‐T) for 1 h at RT with shaking. The membranes were then incubated with 1 µg/mL anti-His-Tag mouse monoclonal antibody (SouthernBiotech) and subsequently with anti-mouse IgG conjugated with alkaline phosphatase (Thermo Fisher) in blocking buffer, each step for 1 h at RT with shaking. After washes, membranes were developed with an nitro blue tetrazolium/5-cromo-4-chloro-indolyl-phosphate (NBT/BCIP) substrate (Thermo Fisher). Following protein expression analysis, cells were harvested and resuspended in lysis buffer (50 mM NaH_2_PO_4_; 300 mM NaCl and 10 mM imidazole) for purification under native conditions. Subsequently, 1 mg/mL lysozyme was added to lysate, which was then incubated on ice for 30 min. The lysate was sonicated on ice six times for 10 s bursts at 200–300W, with a 10-s cooling period between each burst. The lysate was centrifuged at 10.000 *× g* for 30 min at 4°C to pellet the cellular debris. The clarified supernatant was saved and analyzed by SDS-PAGE and WB, as described earlier. Next, 1 mL of ALON Metal Affinity Resin (Takara Bio Company) was mixed with 4 mL clarified lysate by shaking at 4°C for 60 min. The lysate-resin complex was loaded into a column with a capped bottom outlet, and upon removing the bottom cap, the flow-through was collected. Following two wash steps with wash buffer (50 mM NaH_2_PO_4_, 300 mM NaCl, and 20 mM imidazole), the protein was eluted with 0.5 mL of elution buffer (50 mM NaH_2_PO_4_; 300 mM NaCl and 250 mM imidazole) for four cycles. The purified CBM49 was dialyzed against PBS for 48 h, with three consecutive buffer changes and the protein concentration was determined using the BCA kit, as described previously. For CBM49 binding analysis, an ELISA was performed by coating the plates with 50 µL of 10 µg/mL laminarin, curdlan, or dextran as control, following a similar protocol as described previously. After blocking, the CBM49 was serially diluted from 200 to 0.098 µg/mL and incubated with the polysaccharides at RT for 1 h. Upon washes, plates were incubated with 1 µg/mL anti-His-Tag mouse antibodies (SouthernBiotech) and subsequently with anti-mouse IgG conjugated with alkaline phosphatase for 1 h at 37°C. After further washes, plates were incubated with pNPP and absorbances at 405 nm were recorded using a microplate reader.

### Statistical analysis

All statistical analyses were carried out using GraphPad Prism version 9.00 for Windows (GraphPad Software, San Diego, California USA). One-way analysis was used to compare the differences among groups, with a confidence interval of 95% for all experiments, and a *P* < 0.05 was considered statistically significant. Multiple comparisons were conducted using Tukey’s or Dunnett’s correction test, for comparison of every group or to control, respectively, with a single pooled variance.

## Data Availability

The mass spectrometry data that support the findings of this study can be found in the ProteomeXchange online repository (http://www.proteomexchange.org) under the accession number PXD028907.

## SUPPLEMENTAL MATERIAL

Supplementary Figure S1 aem.01736-23-s0001.tif (A) The Filamin repeat domain-containing protein (L8HDD6). Cartoon demonstrating the mapped domains of the signal peptide (black box), Legume Lectin domain (dark blue box), Legume lectin, beta chain, Mn/Ca-binding site (purple diamond), ConA‐like lectins/glucanases superfamily domain (light blue box), Immunoglobulin-like fold (green box), Filamin Family domain (light red), transmembrane helix (light yellow box) and transmembrane domain (dark yellow box) at the C-terminal hydrophobic region of the protein. (B) The carbohydrate-binding domain CBM49 containing protein (L8HAP9) demonstrates the three carbohydrate-binding domains (CBM 49), the carbohydrate-binding type-2 domain, and the two CBM2/CBM3 carbohydrate-binding domains superfamily.

Supplementary Figure S2 aem.01736-23-s0002.tif The coding sequence of the CBM49 containing protein (L8HAP9) was cloned into pET15b plasmid and the recombinant protein was expressed in E. coli. (A) Plasmid map of the pET15b-CBM49opt used for transformation into TOP10 *E. coli* and CBM 49 (L8HAP9) protein expression. (B) Western blot using an anti-His-Tag mouse IgG, as primary antibody and anti-mouse IgG conjugated with alkaline phosphatase as secondary for detection of CBM49 6xHis-tagged protein. Lane 1- Protein molecular weight standard (Precision Plus Protein Standards; BioRad); Lane 2 - noninduced E. coli control. Lane 3 - IPTG-induced cell samples in 1 h; Lane 4 - IPTG-induced cell samples in 2 h; Lane 5 - IPTG-induced cell samples in 3 h; Lane 6 - IPTG-induced cell samples in 4 h; Lane 7 -flow through during purification and Lane 8 - Purified CBM 49 protein.

Supplementary Table S1 aem.01736-23-s0003.xlsx List of proteins identified by Shotgun mass spectrometry proteomics upon purification by affinity to β-1,3-glucans.

Supplementary Table S2 aem.01736-23-s0004.docx Real-time PCR primers used for amplification of the Filamin (L8HDD6, ACA1_149410) and the CBM49 (L8HAP9, ACA1_252830). The 18SQV (18S rRNA gene) was elected as the house-keeping for transcript normalization.

ASM does not own the copyrights to Supplemental Material that may be linked to, or accessed through, an article. The authors have granted ASM a non-exclusive, world-wide license to publish the Supplemental Material files. Please contact the corresponding author directly for reuse.
