# Supplementary figures and images for: β-1,3-Glucan recognition by Acanthamoeba castellanii as a putative mechanism of amoeba-fungal interactions
Source: Appl Environ Microbiol. 2024 Jan 23;90(2):e01736-23. doi: 10.1128/aem.01736-23 (PMC10880599; doi:10.1128/aem.01736-23)

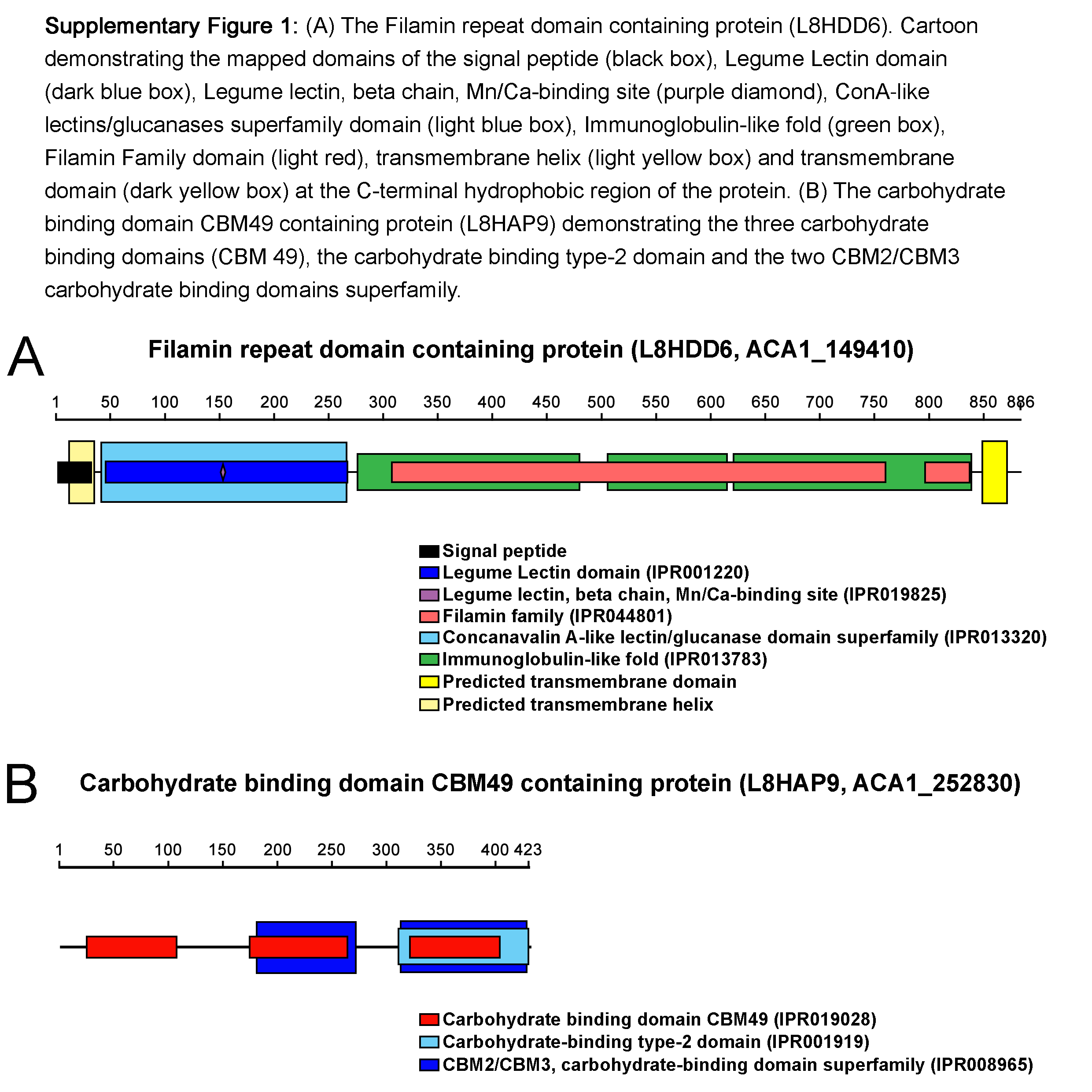

Supplement: Supplementary Figure S1 — (A) The Filamin repeat domain-containing protein (L8HDD6). Cartoon demonstrating the mapped domains of the signal peptide (black box), Legume Lectin domain (dark blue box), Legume lectin, beta chain, Mn/Ca-binding site (purple diamond), ConA‐like lectins/glucanases superfamily domain (light blue box), Immunoglobulin-like fold (green box), Filamin Family domain (light red), transmembrane helix (light yellow box) and transmembrane domain (dark yellow box) at the C-terminal hydrophobic region of the protein. (B) The carbohydrate-binding domain CBM49 containing protein (L8HAP9) demonstrates the three carbohydrate-binding domains (CBM 49), the carbohydrate-binding type-2 domain, and the two CBM2/CBM3 carbohydrate-binding domains superfamily. [file aem.01736-23-s0001.tif]

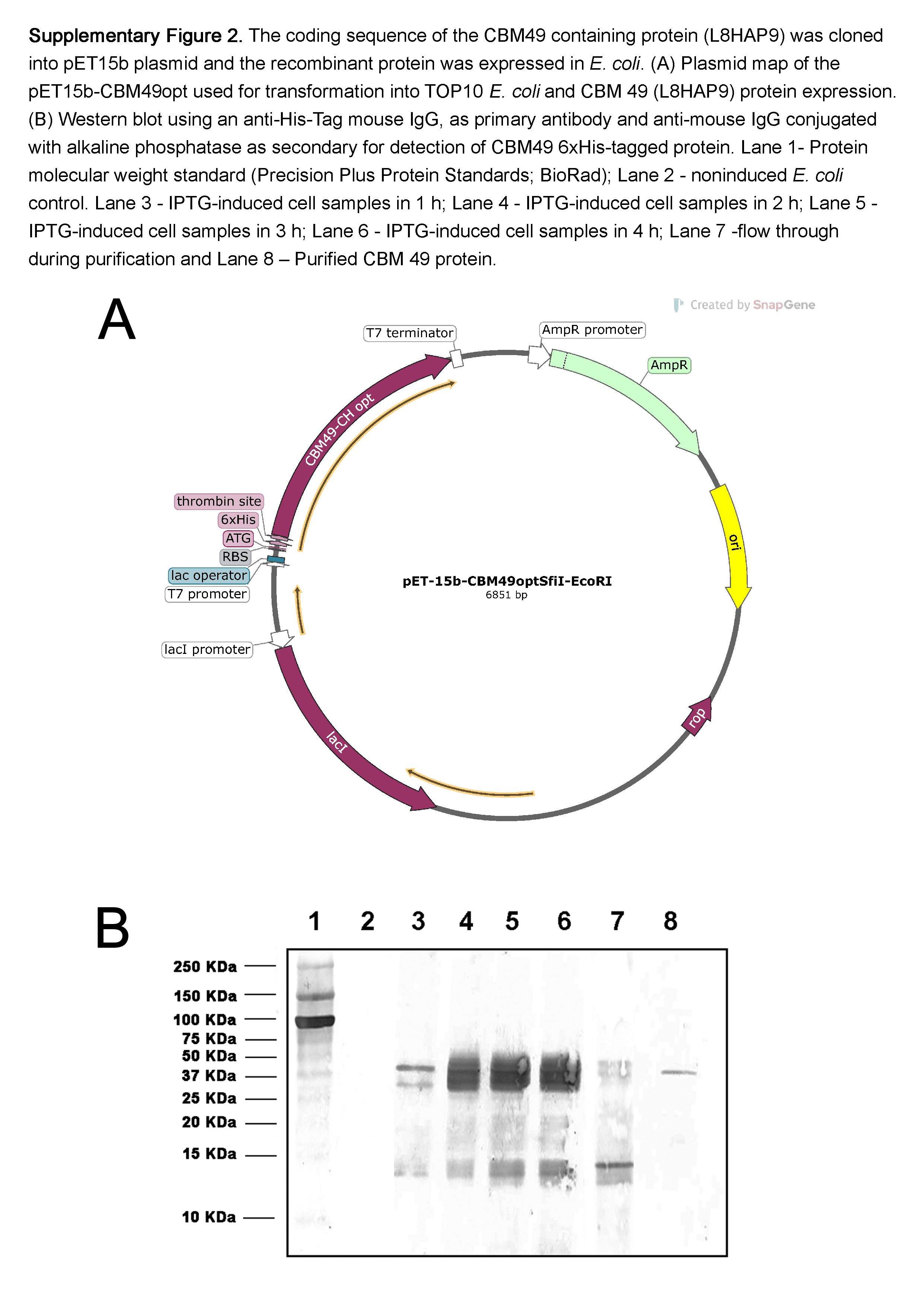

Supplement: Supplementary Figure S2 — The coding sequence of the CBM49 containing protein (L8HAP9) was cloned into pET15b plasmid and the recombinant protein was expressed in E. coli. (A) Plasmid map of the pET15b-CBM49opt used for transformation into TOP10 E. coli and CBM 49 (L8HAP9) protein expression. (B) Western blot using an anti-His-Tag mouse IgG, as primary antibody and anti-mouse IgG conjugated with alkaline phosphatase as secondary for detection of CBM49 6xHis-tagged protein. Lane 1- Protein molecular weight standard (Precision Plus Protein Standards; BioRad); Lane 2 - noninduced E. coli control. Lane 3 - IPTG-induced cell samples in 1 h; Lane 4 - IPTG-induced cell samples in 2 h; Lane 5 - IPTG-induced cell samples in 3 h; Lane 6 - IPTG-induced cell samples in 4 h; Lane 7 -flow through during purification and Lane 8 - Purified CBM 49 protein. [file aem.01736-23-s0002.tif]
