# Supplementary material for: β-1,3-Glucan recognition by Acanthamoeba castellanii as a putative mechanism of amoeba-fungal interactions
Source: Appl Environ Microbiol. 2024 Jan 23;90(2):e01736-23. doi: 10.1128/aem.01736-23 (PMC10880599; doi:10.1128/aem.01736-23)
Supplement: Supplementary Table S2 — Real-time PCR primers used for amplification of the Filamin (L8HDD6, ACA1_149410) and the CBM49 (L8HAP9, ACA1_252830). The 18SQV (18S rRNA gene) was elected as the house-keeping for transcript normalization. [file aem.01736-23-s0004.docx]

**Supplementary Table 2:** Real-time PCR primers used for amplification of the Filamin (L8HDD6, ACA1_149410) and the CBM49 (L8HAP9, ACA1_252830). The 18SQV

(18S rRNA gene) was elected as the house-keeping for transcript normalization.

| **Target** | **Primers** | **Sequences (5’-3’)** | **Length** | **Start** | **Stop** | **Tm (°C)** | **GC%** | **Product length** |
| --- | --- | --- | --- | --- | --- | --- | --- | --- |
| Filamin (L8HDD6) | Forward | GTTCCAACACAACGGCAACC | 20 | 306 | 325 | 60.5 | 55 | 242 |
|  | Reverse | AGGCCGAGTTGGTCTCATTC | 20 | 547 | 528 | 59.8 | 55 |  |
|  |  |  |  |  |  |  |  |  |
| CBM49 (L8HAP9) | Forward | GAGTGCAAGGTGGACATCGTA | 21 | 64 | 84 | 60.1 | 52.4 | 271 |
|  | Reverse | TGGCCTTGCTCTGTGACTTG | 20 | 334 | 315 | 60.5 | 55 |  |
|  |  |  |  |  |  |  |  |  |
| 18SQV  (18S rRNA gene) | Forward | CCCAGATCGTTTACCGTGAA | 20 | 5 | 24 | 60.5 | 50 | 180 |
|  | Reverse | TAAATATTAATGCCCCCAACTATCC | 25 | 184 | 160 | 59.9 | 36 |  |
